# Modeling the distribution of a wide‐ranging invasive species using the sampling efforts of expert and citizen scientists

**DOI:** 10.1002/ece3.5609

**Published:** 2019-09-19

**Authors:** Emilie Roy‐Dufresne, Frédérik Saltré, Brian D. Cooke, Camille Mellin, Greg Mutze, Tarnya Cox, Damien A. Fordham

**Affiliations:** ^1^ The Environment Institute and School of Biological Sciences University of Adelaide Adelaide SA Australia; ^2^ College of Science and Engineering Flinders University Adelaide SA Australia; ^3^ Institute for Applied Ecology University of Canberra Canberra ACT Australia; ^4^ Australian Institute of Marine Science Townsville Qld Australia; ^5^ Department of Primary Industries and Regions South Australia Biosecurity SA Adelaide SA Australia; ^6^ Vertebrate Pest Research Unit NSW Department of Primary Industries Orange NSW Australia

**Keywords:** citizen science, ecological niche model, European rabbit, invasion biology, model transferability, *Oryctolagus cuniculus*, sampling bias, species distribution model

## Abstract

1. In its invasive range in Australia, the European rabbit threatens the persistence of native flora and fauna and damages agricultural production. Understanding its distribution and ecological niche is critical for developing management plans to reduce populations and avoid further biodiversity and economic losses.
2. We developed an ensemble of species distribution models (SDMs) to determine the geographic range limits and habitat suitability of the rabbit in Australia. We examined the advantage of incorporating data collected by citizens (separately and jointly with expert data) and explored issues of spatial biases in occurrence data by implementing different approaches to generate pseudo‐absences. We evaluated the skill of our model using three approaches: cross‐validation, out‐of‐region validation, and evaluation of the covariate response curves according to expert knowledge of rabbit ecology.
3. Combining citizen and expert occurrence data improved model skill based on cross‐validation, spatially reproduced important aspects of rabbit ecology, and reduced the need to extrapolate results beyond the studied areas.
4. Our ensemble model projects that rabbits are distributed across approximately two thirds of Australia. Annual maximum temperatures >25°C and annual minimum temperatures >10°C define, respectively, the southern and northern most range limits of its distribution. In the arid and central regions, close access to permanent water (≤~ 0.4 km) and reduced clay soil composition (~20%–50%) were the major factors influencing the probability of occurrence of rabbits.
5. *Synthesis and applications*. Our results show that citizen science data can play an important role in managing invasive species by providing missing information on occurrences in regions not surveyed by experts because of logistics or financial constraints. The additional sampling effort provided by citizens can improve the capacity of SDMs to capture important elements of a species ecological niche, improving the capacity of statistical models to accurately predict the geographic range of invasive species.

## Graphical abstract

We developed an ensemble of species distribution models (SDMs) to determine the geographical range limits and habitat suitability of an important invasive species, the European rabbit, in Australia. We examined the advantage of incorporating data collected by citizens (separately and jointly with expert data) and explored issues of spatial biases in occurrence data by implementing different approaches to generate pseudo‐absences. Our results show that citizen science data can play an important role in managing invasive species by providing missing information on occurrences in regions not surveyed by experts, improving the capacity of SDMs to capture important elements of a species ecological niche and therefore their ability to predict the structure of the geographic range of invasive species.

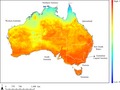

## 1 INTRODUCTION

The spread of invasive species across the world is a major driver of current observed ecosystem changes (Lowry et al., 2013). Such changes include damage to native habitats (Simberloff et al., 2012) and biodiversity loss (Mollot, Pantel, & Romanuk, 2017), leading to important socioeconomic costs (McLeod, 2004). Many strategies are proposed to quantify the risk associated with the introduction and the spread of invasive species (e.g., Mack et al., 2000). Most suggest the use of species distribution models (SDMs) as a tool to model the habitat suitability of invasive species with an objective to predict and prevent invasion events (Thuiller et al., 2005).

Two main approaches are used for modeling the structure and dynamics of the geographic ranges of invasive species (Robertson, Peter, Villet, & Ripley, 2003). Mechanistic‐based distribution models use inherent physiological and/or demographic characteristics to better capture the processes underpinning species distributions (Fordham, Akçakaya, Araújo, Keith, & Brook, 2013; Kearney & Porter, 2004). Correlative‐based distribution models use a different approach by linking invasive species observations to environmental conditions (e.g., climate and vegetation) using statistical techniques (Guisan & Thuiller, 2005). Correlative approaches (SDM herein) remain the most frequently used methods for exploring the determinants of the range of invasive species and their probability of occurrences due to simpler data requirements (Elith, Kearney, & Phillips, 2010).

Robust predictions from SDMs require the models to be trained using data (i.e., presence/absence field observations) obtained from the entire range of environmental conditions suitable for the persistence of the species (Elith et al., 2010). Gathering these data is challenging for invasive species since they (a) are often not in an equilibrium‐state with their host environment (Sutherst & Bourne, 2009); (b) can exhibit opportunistic behaviors allowing them to survive and reproduce under conditions differing from their native ranges (Mellin et al., 2016); and (c) are often widely distributed in their nonnative range making the data collection process time‐consuming, costly, and logistically challenging (Hauser, Pople, & Possingham, 2006). To overcome this difficulty, data collected by experts can be supplemented with data collected by volunteers commonly referred to as citizen scientists (Silvertown, 2009). Doing so broadens the sampling effort spatially and temporally, potentially improving the projections of invasive species' distributions in their novel habitat (Dickinson, Zuckerbert, & Bonter, 2010).

Integrating citizen science data into SDMs can generate methodological challenges. For instance, sampling biases may need to be explicitly accounted for in the models due to volunteers frequently collecting data in opportunistic and subjective ways (e.g., during recreational activities in areas easy to access and with important natural attractions; Fourcade, Engler, Rödder, & Secondi, 2014). These sampling biases can both inflate the species' presence in localized areas and cause some environmental habitats to be overlooked (Crall et al., 2010; Fitzpatrick, Preisser, Ellison, & Elkinton, 2009), increasing the likelihood of type 1 errors from models (Hanspach, Kühn, Schweiger, Pompe, & Klotz, 2011), generating misleading predictions (Osborne & Leitão, 2009).

In this study, we asked whether citizen science data could be used in SDMs to generate robust predictions of the distributions of a wide‐ranging invasive species: the European rabbit (*Oryctolagus cuniculus*) in Australia. The species was introduced into the country in 1788 and is listed as a Key Threatening Process under the Environment Protection and Biodiversity Conservation Act since 1999 due to competition with the native fauna and flora and overgrazing activities (West, 2008). Over the last 50 years, rabbit occurrence and abundance have been monitored by expert scientists under various governmental and local programs across the country (Roy‐Dufresne, Lurgi, et al., 2019; Roy‐Dufresne, Saltré, et al., 2019). In 2009, a citizen science initiative was launched to record sightings of rabbits across Australia (Feral Scan Data, 2016).

We (a) examined the advantages of incorporating data collected by citizens in SDMs (separately or jointly with expert data) to pinpoint areas of high environmental suitability for rabbits in Australia; (b) explored potential issues of spatial biases in citizen and expert occurrence data, which we addressed by implementing different approaches to generate pseudo‐absences; and (c) produced a high‐resolution map of habitat suitability in support of pest management activities. Our results show the important role that citizen science data can play in invasive species management by providing missing information on environmental–occurrence relationships in regions not surveyed by experts, improving the fit of SDMs.

## 2 MATERIALS AND METHODS

### 2.1 Occurrence records and environmental covariates

**Figure 1:**
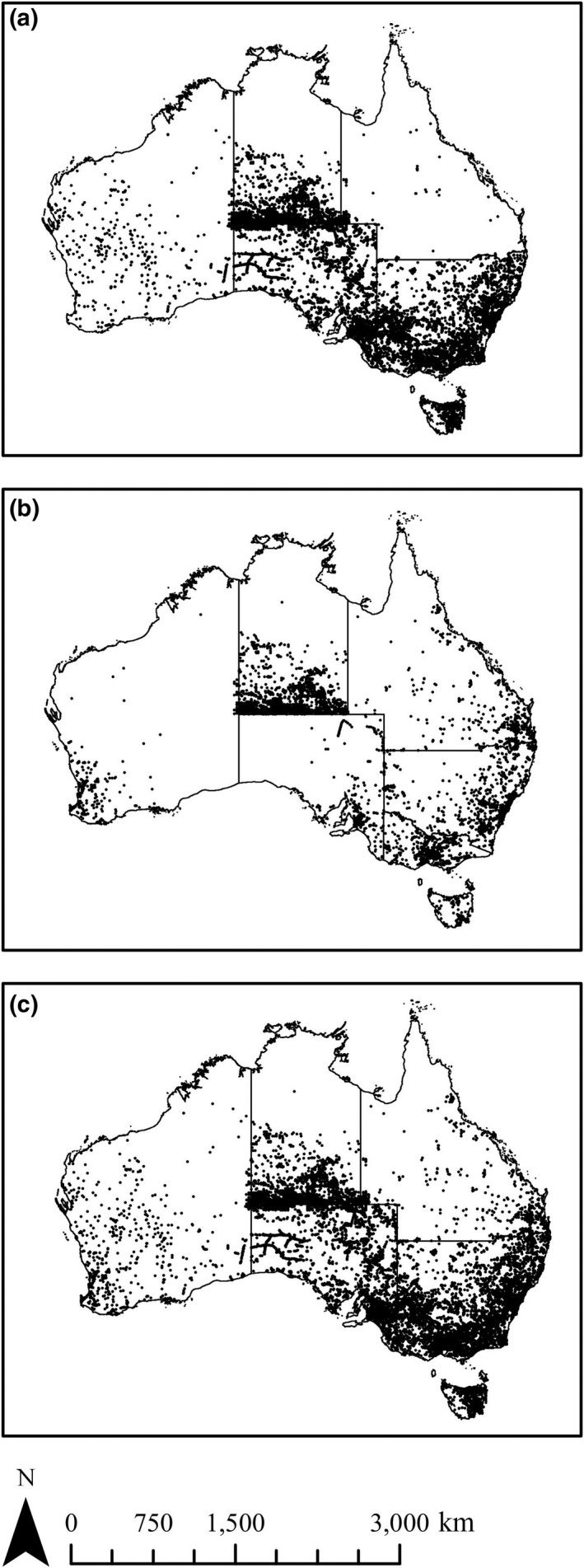
Distribution of *Expert* (a), *Citizen* (b), and *Combined* (c) rabbit occurrences (black dots) in Australia

Rabbit's occurrences by experts were collated from (a) the Tasmanian Natural Values Atlas (Department of Primary Industries, Parks, Water, & Environment, 2016), (b) the Victorian Biodiversity Atlas (Department of Environment, Land, Water, & Planning, 2016), (c) the Nature Map from Western Australia (Department of Parks & Wildlife, 2016), (d) the Fauna Atlas of the Northern Territory (Northern Territory Government, 2016), (e) the NSW Office of Environment Heritage Atlas of Wildlife (Department of Environment & Heritage, 2016), and (f) the Atlas of Living Australia (Atlas of Living Australia, 2016). These records were supplemented using occurrence data from the national rabbit database (Roy‐Dufresne, Lurgi, et al., 2019; Roy‐Dufresne, Saltré, et al., 2019) (total: 3,409 pts). *Citizen* occurrences were obtained from the Feral Scan surveillance program on 05‐21‐2015 (Feral Scan Data, 2016; total: 1,842 pts). *Combined* occurrences were obtained by merging the citizen and expert data (total: 4,011 pts). Occurrence records were constrained to the period from 1970 to 2012 to match the temporal period covered by the environmental covariates (see below). All records were mapped at a 1‐km^2^ grid cell resolution and verified using expert knowledge to remove erroneous occurrences (i.e., those situated outside the known biophysical limits of the rabbit in Australia) as suggested by Drescher et al. (2013; Figure 1).

We used published literature to initially select 15 environmental covariates (e.g., climate, vegetation, and soil) likely to influence the occurrence of rabbit in Australia (Supporting Information S1). Covariates were obtained in a grid format at 1‐km^2^ grid cell resolution for Australia and were projected to the same geographic reference system (i.e., WGS84). Some covariates were transformed (Supporting Information S1) to better meet the assumptions of our statistical models (see below; Austin, 2002). We tested for collinearity (Zuur, Ieno, & Elphick&, 2010) using the Spearman rank correlation coefficient (*Hmisc* package in R; Harrell, 2016; R Development Core Team, 2017) and the variance of inflation factor (vif; using *car* Package in R; Fox & Weisberg, 2011). We excluded highly correlated (i.e., Spearman's Rank *r* ≥ ±0.7) and collinear (i.e., vif ≥ 3) covariates from further analysis in favor of covariates likely to be more ecologically relevant in explaining the distribution of rabbits in Australia. This resulted in seven primary covariates being used in the analyses (Table 1).

**Table 1:** Name, description, and range of value of selected covariates to describe the distribution of the rabbits in Australia

Covariates name	Description	Range of value
*TMin* a	Mean annual minimum temperature (°C) between 1976 and 2005	−5.5; 24.5
*TWarmestMonth* a	Mean annual temperature of the warmest month (°C) between 1976 and 2005	8.1; 33.3
*PWetQuarter* a	Mean total precipitation of the wettest quarter (mm; log‐transformed)	3.7; 8.0
*VegeType* b	Thirteen categories of major vegetation groups (reclassification described in Supporting Information S1)	1; 13
*DistAgriLand* c	Euclidean distance (km) to the nearest agricultural land margins (square root)	0; 31.8
*DistPermWater* d	Euclidean distance (km) to nearest permanent water features and surface hydrology points (square root)	0; 14.6
*PercSoilClay* e	Median percentage of clay (log‐transformed)	−1.7; 4.1

Note

See Supporting Information S1 for the ecological reasons.

a Hutchinson, Kesteven, and Xu (2014),

b Department of the Environment (2012),

c Lymburner et al. (2010),

d Geoscience Australia (2006, 2015),

e Northcote et al. (1991).

### 2.2 Spatial autocorrelation and pseudo‐absences

Because we only had access to occurrence records, we generated pseudo‐absences to calibrate the SDMs using two strategies and compared their statistical support. These strategies were as follows: (a) weighting the location of the pseudo‐absences according to the density of the occurrence data (*Weighted Pts*), and (b) generating pseudo‐absences randomly (*Random Pts*).

Pseudo‐absence strategy *Weighted Pts* accounted for potential sampling bias in rabbit occurrences by positively weighting their selection probability using a proxy of sampling effort (Syfert, Smith, & Coomes, 2013). More specifically, we generated the pseudo‐absences using a similar sampling bias configuration to the occurrence data (Phillips & Dudík, 2008). A proxy for sampling effort was obtained from the density of the occurrence data at 1‐km^2^ grid cell resolution (*spatstat* package in R; Baddeley, Rubak, & Turner, 2015). The robustness of the resulting grids was tested using Ripley's L‐function (*spatstat* package in R; Baddeley et al., 2015), which assessed the spatial homogeneity of the data (i.e., random, dispersed, or clustered) in comparison with what would be expected from a random uniform distribution. The results from this analysis (Supporting Information S2) showed that we could simulate a similar level of sampling densities in our pseudo‐absence data as that observed from each occurrence dataset. For comparison, pseudo‐absences were also generated using a random strategy (i.e., without accounting for sampling effort; *Random Pts*; Wisz & Guisan, 2009). The number of pseudo‐absences generated, for both strategies, was set to four times the number of occurrence points, providing maximal coverage of the study area as suggested by Barbet‐Massin, Jiguet, Albert, and Thuiller (2012).

Spatial autocorrelation is a common issue in ecological data and can exaggerate the importance of explanatory covariates. We used a Moran's I index (Global Moran's I function in ArcMap 10.3.1; ESRI, 2015) to test for spatial autocorrelation in each occurrence dataset. We determined the spatial resolution that minimized spatial autocorrelation without compromising the ecological relevance of each occurrence dataset (i.e., by choosing lower spatial resolution possible; Dormann et al., 2007). We compared the distribution of a set of points randomly distributed over the study area (10 times the number of occurrence data) with the distribution of our datasets aggregated at different resolutions (i.e., 1, 5, 10, 20, 30, 50, 70, 100, 150, and 200 km). The Moran's I analysis showed that spatial autocorrelation in the occurrence datasets was best controlled at a 20‐km resolution (Supporting Information S3). We resampled the occurrence and pseudo‐absence datasets by taking one point per 20 km^2^ grid cell. We repeated the sampling exercise until every occurrence was selected at least once, giving a total of 105 replicates per dataset. To take into account different occurrence–environmental relationships, we ran all further analysis at the replicate level and then calculated the across replicate mean value (Araújo & Guisan, 2006).

### 2.3 Model construction and evaluation

We used three common correlative SDM algorithms to model the distribution of rabbits in Australia: (a) general linear models (GLM; regression approach without interaction and including quadratic functions), (b) Boosted Regression Trees (BRT; ensemble of regression trees), and (c) a Maximum Entropy algorithm (MaxEnt; machine learning approach). The GLMs were parameterized using a logit‐link function and a binomial error distribution. The BRT models were fitted using the *gbm* package in R (Ridgeway, 2017). We used a ten‐fold cross‐validation to identify the optimal settings by systematically altering the different combination of numbers of trees (100–10,000 at a 100 interval), learning rates (0.0001, 0.005, 0.001, 0.005, 0.01), and tree complexities (1–5). Based on the difference between the observed and predicted values of those combinations, we selected the setting returning the smallest deviance, number of trees, and tree complexity (Elith, Leathwick, & Hastle, 2008). We fitted the MaxEnt models (package *dismo* in R; Hijmans, Phillips, Leathwick, & Elith, 2017) using all six data transformation features available within MaxEnt (i.e., linear, product, quadratic, hinge, threshold, and categorical) and by specifying background data points using predefined pseudo‐absence datasets. The regularization coefficient values were maximized from a combination of values (0.2–5 at a 0.2 interval) based on a 5‐fold cross‐validation process.

To determine the “best” model and to avoid over‐parametrization, we first constructed a set of candidate models based on expert knowledge, representing different biological processes (and their combination) likely to define the rabbit distribution in Australia (Supporting Information S4). We used a two‐phased analytical approach to select the best model (e.g., Wadley, Austin, & Fordham, 2014). We first constructed a candidate set of models with only climatic and another with nonclimatic covariates and used multimodel inference to select the best models for each group. We ranked the models using the Akaike's information criterion corrected for small sample size (AIC_c_) and assessed their probability relatively to the entire set of candidate models using the AIC_c_ weights (wAIC_c_) and their corresponding percentage of deviance explained (Burnham & Anderson, 2010). In step 2, we generated a separate candidate model set with all potential combinations of covariates from the best‐ranked models (wAICc = 1) in step 1. We did this preliminary analysis only with the expert occurrence data, which is more precise and reliable (Roy‐Dufresne, Lurgi, et al., 2019; Roy‐Dufresne, Saltré, et al., 2019) and, therefore, provides a better reflection of the pattern of occurrence for the focal species.

We evaluated the models performance using two approaches: a cross‐validation analysis and an out‐of‐region validation analysis. The first approach evaluated the models predictive ability by repeating 5‐fold cross‐validation in which the occurrence data were randomly partitioned into a training and test sets of respectively (80%/20% ratio; Fielding & Haworth, 1995). The out‐of‐region approach allowed us to evaluate the models' transferability across regions (Randin et al., 2006). For this analysis, we selected the physiographic regions of Australia, which are geomorphological units coherent with the landform characteristics and the underlying geology (Pain, Gregory, Wilson, & McKenzie, 2011). They provide a basic differentiation of soil types and natural vegetation, which are important factors determining the availability of shelter and food resources for rabbits (Myers & Parker, 1975). Successively, we used the occurrence data from each region as the test set, while calibrating the models with the data from the remaining regions (i.e., training set).

We used the area under the receiver operating characteristics curve (AUC) (Jiménez‐Valverde, Acevedo, Barbosa, Lobo, & Real, 2012), and the Kappa statistic (Manel, Williams, & Ormerod, 2001), as metrics of models' performance. To extract the Kappa score, we calculated a prevalence threshold by maximizing the sum of the sensitivity and specificity (Manel et al., 2001), while he AUC does not require a threshold to be defined (Jiménez‐Valverde et al., 2012).

We (G.M., B.D.C., and T.C.) visually evaluated the response curves of the models using our extensive knowledge of rabbit ecology in Australia (e.g., Syfert et al., 2013; Supporting Information S5). Values from 0 to 3 (i.e., poor to excellent representation of ecological reality) were assigned to each response curve (Supporting Information S5). We weighted these values by a standardized estimate (scaled between 0 and 100) of the importance of all covariates in the models and took the average result.

Covariate importance was calculated for each SDM algorithm using model‐specific approaches. For the GLMs, we used a paired *t*‐statistic to test for covariate importance before and after permuting the value of one covariate and keeping the values of the other covariates constant (Ridgeway, 2017). For the BRTs, we calculated the number of times covariates were selected for splitting the trees, weighted by the squared improvement of the models fit as a result of each split, averaged over all trees (Ridgeway, 2017). For MaxEnt models, we changed the values of each covariate across its range values obtained from the training occurrence set and measured the resulting decrease in the AUC value (Hijmans et al., 2017).

### 2.4 Mapping probability of occurrence

To map the probability of rabbit occurrence in geographic space, we used an ensemble modeling approach. This is because evidence from various areas of numerical modeling suggests that multimodel averages often yield better predictions than a single model (Johnson & Omland, 2004). Weighted averaging of different SDM results is now widely used to account for model‐selection uncertainty under the assumption that this will lead to more robust estimates of model predictions of probability of occurrence (Araújo & New, 2007). We calculated the ensemble projection using the best‐ranked models for each of the three occurrence datasets. We calculated the average values of these models weighted according to their cross‐validation Kappa scores (Araújo & New, 2007). We used the Kappa scores to calculate the weighted ensemble projection because it is a more rigorous test of model skill than AUC scores (Jiménez‐Valverde, 2011), resulting in more variability in model evaluation scores.

## 3 RESULTS

### 3.1 Model parameters and pseudo‐absences generation

The global model (i.e., with all environmental covariates based on a subset of good performing climatic and nonclimatic models; see Methods) had the greatest AICc support (wAIC_c_ > 0.79, Mean Explained Deviance >27%; Supporting Information S4), irrespectively of the pseudo‐absences' strategy used. There was one notable exception: BRT with weighted pseudo‐absences supporting a simpler model that did not include vegetation type (wAIC_c_ = 0.66) compared to the global model (wAIC_c_ = 0.33), but the mean explained deviance was essentially the same (~27%).

**Figure 2:**
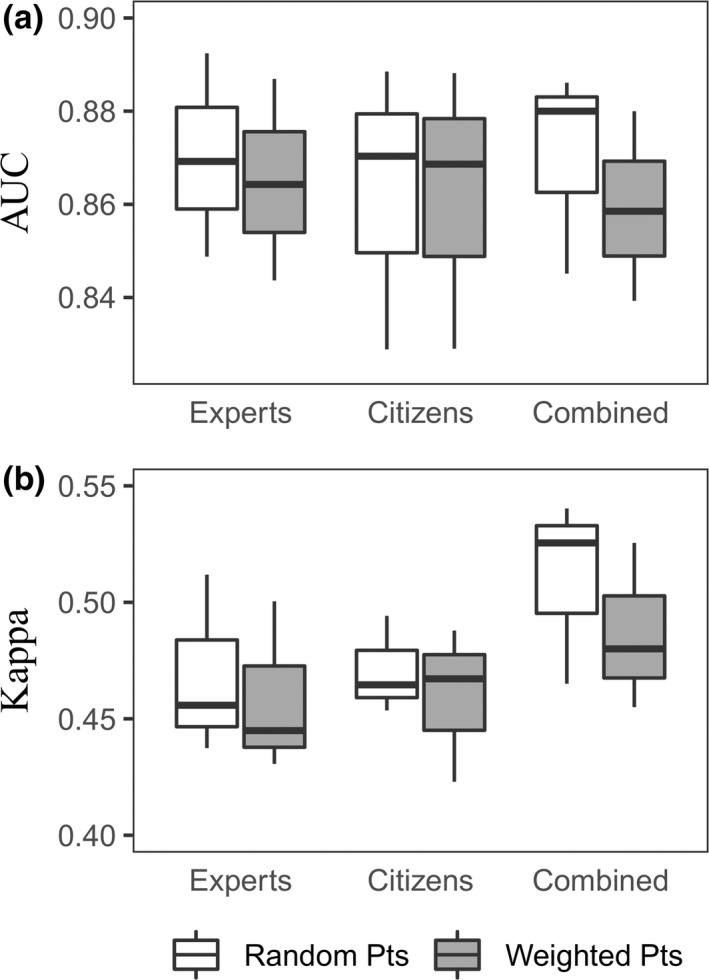
Boxplots of area under the receiver operating characteristics curve (AUC; a) and Kappa (b) cross‐validation scores for species distribution models based on *Expert*, *Citizen*, and *Combined* datasets and pseudo‐absences based on *Random Pts* and *Weighted Pts*. The central mark indicates the median, and the bottom and top edges of the box indicate the 25th and 75th percentiles, respectively. The whiskers extend to the most extreme data points not considered outliers

**Figure 3:**
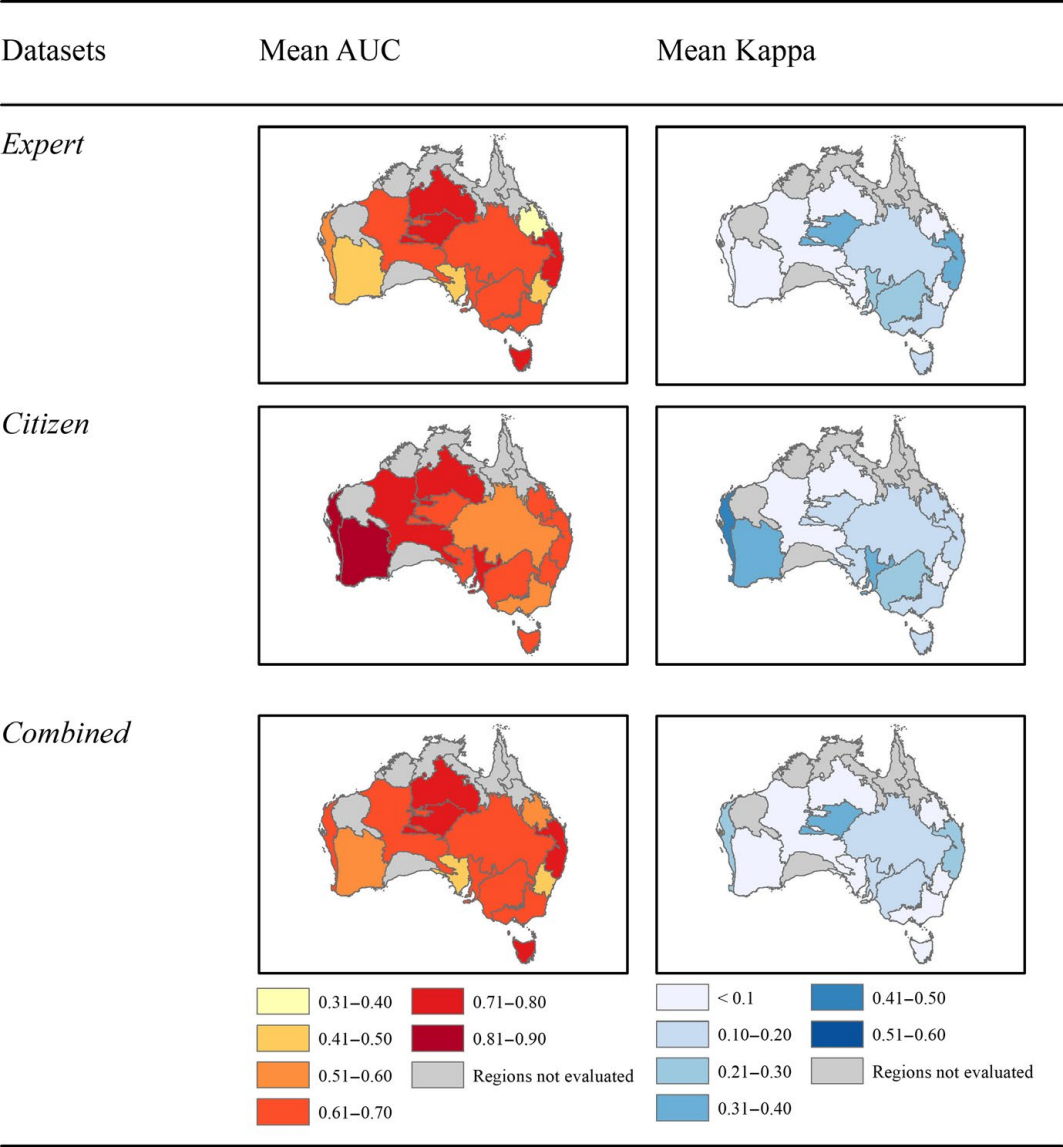
Area under the receiver operating characteristics curve (AUC) and Kappa results from the out‐of‐regions analyses based on three different occurrence datasest (*Expert*, *Citizen*, and *Combined*) and pseudo‐absences based on *Random Pts*. The figures were obtained by taking the mean of the results across all algorithms. The land divisions represent the locations of the physiographic regions of Australia and the regions in gray were not evaluated due to too lower number of occurrence points (*n* < 25). The results for the *Weighted Pts* pseudo‐absence strategy are provided in the Supporting Information S6

Models built using pseudo‐absences generated with the *Random Pts* strategy had cross‐validated AUC and Kappa scores higher than models using the *Weighted Pts* strategy (max ΔAUC = 0.022 and max ΔKappa = 0.045; Figure 2; Supporting Information S6). Likewise, based on out‐of‐region model validation, there was more support for the *Random Pts* method. Models with randomly generated pseudo‐absences predicted well in 43 regions based on AUC values >0.7 (across all algorithms and datasets), and in 4 regions based on Kappa values >0.4, indicating a “fair” model transferability in these regions (Figure 3; Landis & Koch, 1977; Thuiller et al., 2005). This is compared to 42 and three regions, respectively, for models with *Weighted Pts* methods (Supporting Information S6). Using the *Weighted Pts* strategy did not improve the ecological robustness of the response curves based on expert assessment when compared to the *Random Pts* strategy (mean Δscores for *Expert* model = 1.10, *Citizen* model = 0.7, and *Combined* model = 0.47; Supporting Information S6).

Since the results from the three different evaluation techniques provided consistent support for building models with randomly generated pseudo‐absences (i.e.,* Random Pts*), we focused only on the results from these models in the following sections. The *Weighted Pts* results can be found, for comparative purposes in the supplementary material (Supporting Information S6).

### 3.2 Expert versus citizen versus combined data

The cross‐validated analyses showed different results according to different evaluation metrics. There was little difference in AUC validation scores regardless of the source of data (*expert*, *citizen*, *combined*) used to calibrate the model (Figure 2; Supporting Information S6). All models obtained a mean AUC score >0.82 indicating a “fair” fit to the occurrence data (see above). In contrast, Kappa scores indicated that models built with *Combined* datasets (mean Kappa = 0.51, *SD* = 0.03) had better cross‐validated predictive accuracy compared to models calibrated with *Expert* (mean Kappa = 0.47, *SD* = 0.03) or *Citizen* datasets (Kappa = 0.47, *SD* = 0.02; Figure 2).

The out‐of‐region analyses showed that models built with the *Citizen* occurrence data had more regions consistently with higher Kappa and AUC scores (Figure 3; Supporting Information S6). Predictions from models trained with the *Combined* data tended to have lower AUC and Kappa values. The scores obtained for each region were, however, always in close proximity to the scores obtained from the better ranked models with *Citizen* data (mean ΔAUC = 0.05, *SD* ΔAUC = 0.11; mean ΔKappa = 0.06, *SD* ΔKappa = 0.13; Supporting Information S6). Larger differences between out‐of‐region validation scores were observed between models calibrated with *Expert* or *Citizen* datasets (mean ΔAUC = 0.14, *SD* ΔAUC = 0.11; mean ΔKappa = 0.13, *SD* ΔKappa = 0.14). Models using *Citizen* data predicted well into 18 regions based on AUC (>0.7) and two regions based on Kappa (>0.4), while models using Expert and Combined data predicted well into 13 and 12 regions based on AUC and 2 and 0 regions based on Kappa, respectively (Figure 3). More generally, models trained using *Citizen* data had better predictive capacity in the western and central regions of Australia, while for models trained with *Combined* and *Expert* data, predictions were better in the eastern regions (Figure 3).

The response curves from models trained using *Expert* or *Combined* data had similar ranks based on expert knowledge (from 31.01 to 40.34 and 28.97 to 39.37, respectively), whereas models trained with *Citizen* data had lower rankings (scores 21.08–39.72; Supporting Information S6–S7).

### 3.3 Important covariates

**Figure 4:**
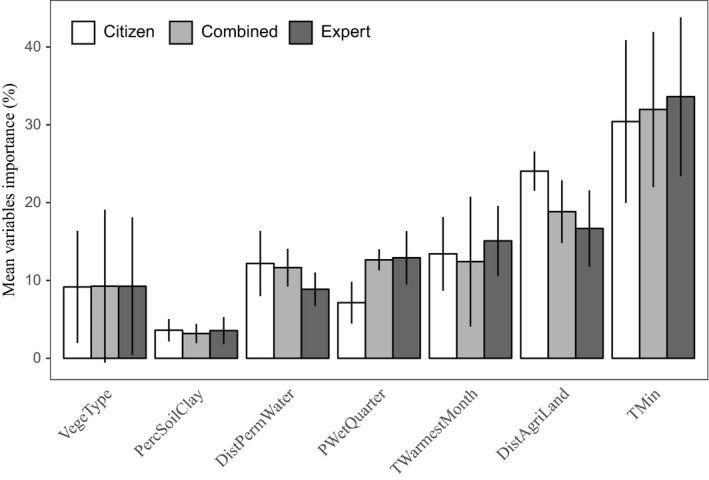
Mean covariates importance (%) and their corresponding standard deviations (line range) for the *Random Pts* pseudo‐absence strategy based on three different sources of dataset (*Expert*, *Citizen*, and *Combined*)

The most important covariate for determining rabbit occurrence was *TMin* (mean importance score of 32%), followed by either combination of *DistAgriLand* (importance score of 19.85%) or *TWarmestMonth* (mean importance score of 13.64%; Figure 4). The next most important covariates were *PWetQuarter* and *DistPermWater* with mean importance scores of 10.91% and 10.90%, respectively. The covariates *PercSoilClay* and *VegeType* had the lowest contributions (importance score <10%). The variables are described in Table 1.

Models calibrated with *Expert* data assigned a stronger importance to the covariate *TWarmestMonth* and lower importance to the covariate *DistPermWater* than models using *Citizen* or *Combined* data (Figure 4). Conversely, models with *Citizen* data gave greater importance to the covariate *DistAgriLand* and less to the covariate *PWetQuarter* then models with *Expert* or *Combined* data.

### 3.4 Probability of occurrence across Australia

**Figure 5:**
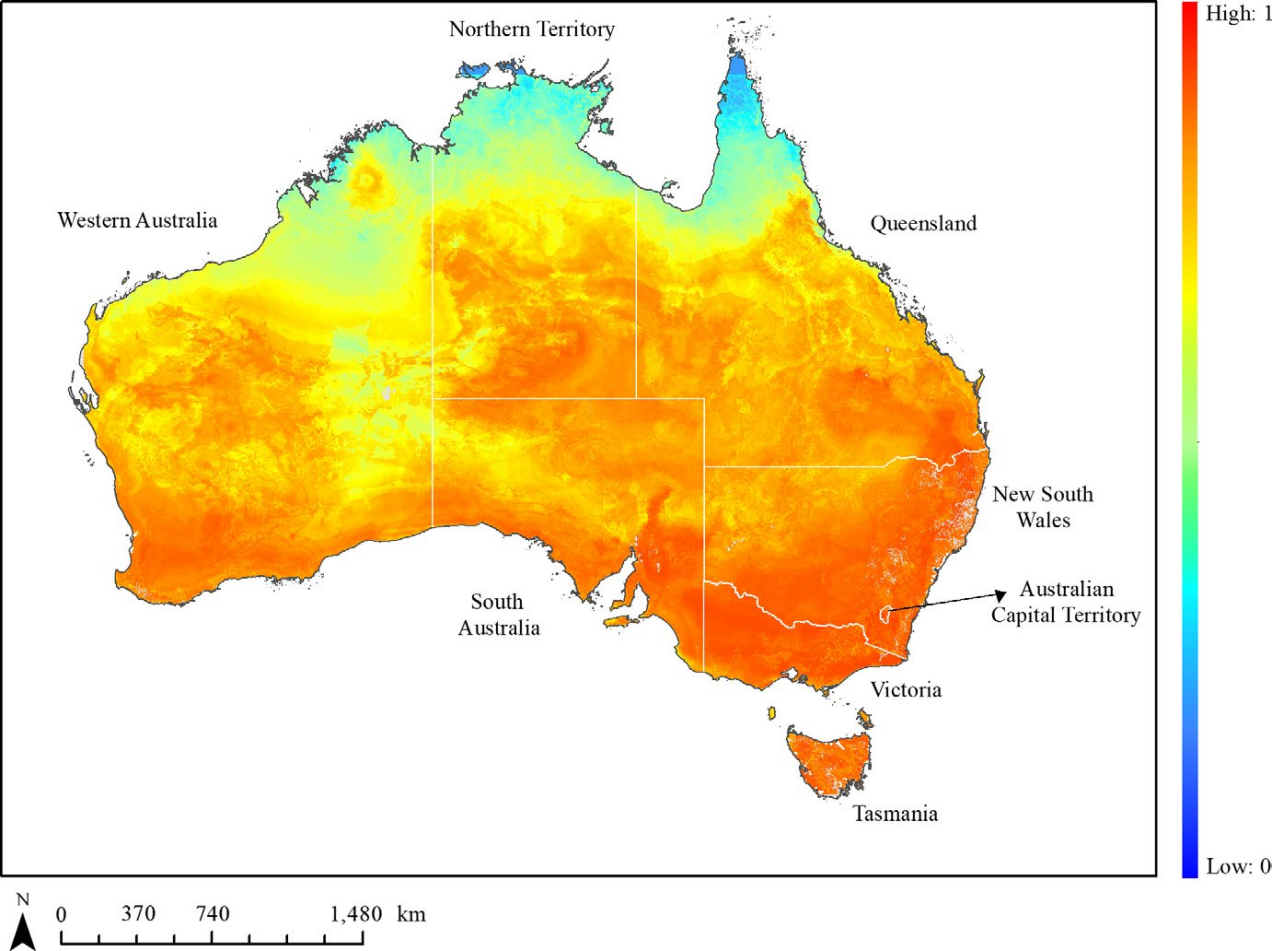
Ensemble averaged probability of occurrence of rabbits across Australia. Gradient goes from dark blue (probability 0) to bright red (probability of 1). The white land divisions and the dotted lines represent the location of state boundaries in Australian. The light gray regions are NA value resulting from missing information for some of the covariates

Our ensemble model (with combined occurrence data with Random Pts pseudo‐absence strategy; Supporting Information S8) showed that regions of Australia south of the 32nd parallel are suitable for rabbit occupancy (scores >0.75; Figure 5), that is where *TWarmestMonth* <25°C. The deserts and regions above the Tropic of Capricorn (19th parallel south) are inadequate for rabbit occupancy (score <0.25; *TMin* > 10°C), with the exception of northern parts of Western Australia and the north‐eastern part of Queensland. In the arid and central regions of Australia, the probability of occurrence of the species ranges between 0.6 and 0.9 with higher scores in regions in close distance to permanent water (<~0.4 km) and with sandy loam soil substrate (20%–50%).

## 4 DISCUSSION

### 4.1 Including citizen data in SDMs

The use of citizen data in SDMs is often criticized due to uncertainties associated with underlying sampling processes (Mair & Ruete, 2016). Our results, however, highlight a number of important advantages of using citizen data in ecological models for wide‐ranging invasive species. Using *Citizen* data, in addition to *Expert* data, doubled the spatial coverage of our occurrence data (i.e., ~1/3 Australian land surface), providing (a) new and important information on the environmental conditions associated with the occurrence of rabbits in Australia; and (b) improved model predictions based on cross‐validation and out‐of‐region validation.

The results obtained from the out‐of‐region transferability analyses showed distinct geographic differences in transferability between the data sets used to calibrate the model. Models calibrated with *Citizen* data were more skillful in projecting into the western and central physiographic regions, while models calibrated with *Expert* and *Combined* data did better in Eastern regions of Australia. These contrasting results most probably reflect difficulties in extrapolating to novel conditions and bring attention and awareness to the underlying issues associated with model transferability (Qiao et al., 2018). As the novelty of the environmental conditions in the region being evaluated increases, the model transferability performance scores decrease (Sequeira et al., 2016) while the probability of obtaining erroneous predictions increases (Elith & Leathwick, 2009).

From Figure 1, we can see that the geographic variation in the out‐of‐region cross‐validation scores reflect the spatial variation in the locations of the *Expert* and *Citizen* data, that is regions attained better performance scores with models using *Expert* data where more *Expert* data were collated, and vice‐versa for the *Citizen* data. Since *Citizen* data were present in more regions than *Expert* data, models calibrated with those data were more transferable across the study area (Figure 3). These results highlight the importance of training SDMs with data obtained from the entire species' range (Elith et al., 2010), which in this case, was achieved using Citizen data.

We would have expected models with *Combined* data to obtain the best scores for the out‐of‐regions analyses, since they require the least amount of extrapolation. However, they had slightly poorer transferability scores. This is because leave‐one‐region‐out validation was used to assess model performance, and a larger number of occurrences in the *Combined* dataset in the validation region meant that the analytical test was more rigorous in most regions, by virtue of a greater number of validation points. Using a fixed number of independent occurrences for every region would have provided a better evaluation dataset for the comparative analysis, but we did not have such a dataset.

### 4.2 Accounting for sampling bias in SDM training

Although issues regarding sampling processes are usually associated with citizen science data (Reddy & Dávalos, 2003), in our study *Expert* data showed important spatial biases. The biases in the *Expert data* are likely due to many years of research around the same study sites, for which locations were selected to answer specific research questions (e.g., assessing benefits of rabbit control methods) or for logistical reasons (e.g., easy to access sites; and sites with sufficient numbers of rabbits for sampling).

Generating pseudo‐absences weighted positively to the density of the occurrence data (following Phillips & Dudík, 2008), did not improve the skill of the models. Similar results were obtained by Syfert et al. (2013) and Tye, McCleery, Fletcher, Greene, and Butryn (2016) and were attributed to the bootstrapping method used to evaluate model performance (Phillips et al., 2009). Since both the test and training datasets are sampled from the same initial set of data, they are similarly biased, resulting in evaluation scores indistinguishable from models with random pseudo‐absences. Using an independent set of occurrence data which do not suffer from sampling bias as test data would potentially provide a better assessment of the correction method proposed here (Loiselle et al., 2008), but again such a dataset was not available.

### 4.3 Rabbit biogeography in Australia

Our ensemble model projects that the environmental conditions suitable for rabbit persistence covers more than two third of the country, with the highest levels of probability of occurrence being in the southern regions of Australia below the Tropic of Capricorn (23rd parallel south) except for areas such as north‐eastern Queensland, where rabbits extend toward 19th parallel south. This wide spatial distribution is supported by other studies (Fordham, Akçakaya, Araújo, & Brook, 2012; West, 2008). Our approach, however, provides more detailed descriptions of the rabbit's distribution based on climatic and nonclimatic covariates and is modeled at a much finer spatial resolution more relevant to the species biology.

Mean temperature of the warmest month (*TWarmestMonth*) and mean annual minimum temperature (*TMin*) had the greatest influence on probability of occurrence, regardless of the data set used to calibrate the model. In southern regions of Australia, where *TWarmestMonth* is <25°C, the highest probabilities of occurrence (i.e., >0.85) were observed, while the reverse trend was obtained for the arid northern regions of Western Australia including the deserts where *TWarmestMonth* >28°C (i.e., <0.6). Although the species biology is complex, temperatures >25°C are often reported to stress rabbits, causing reproductive rates to decline (Cooke, Brennan, & Elsworth, 2018). Similarly, regions with TMin > 10°C have low probabilities of occurrence (i.e., <0.4). In these regions rabbits are unlikely to escape the stress exerted by the heat and humidity even when hiding in warrens during the day (Myers & Parker, 1975).

In the arid and central regions of Australia, rabbit populations are more likely to be observed near landscape structures which could provide adequate food resources and sheltered protection against the heat (Figure 5). Although rabbits primarily rely on the water content of the plants they consume (Berman, Brennan, & Elsworth, 2011; Cooke, 1982), rabbits do drink during severe drought. Furthermore, permanent water may also be associated with surrounding vegetation with higher water content and therefore aid survival during droughts (e.g., distance to permanent water <~0.4 km). Generally, the probability of occurrence of rabbits is also influenced by soil‐type (e.g., 20%–50% of soil that is clay) which not only explains warren distribution (Myers & Parker, 1975) but also the persistence of perennial food plants across the year (Berman et al., 2011).

Although the ensemble model overestimated the known current distribution of the rabbit in some regions of Australia, such as the north of the Northern Territory (e.g., Tanami desert and Barkly Tablelands) and some regions in South Australia (e.g., the Victoria Desert region and Pinkawillinie National Park), these same regions are characterized with occasional and widespread sightings of rabbits by citizens (West, 2008). This raises concern about the low level of monitoring in areas where the species might establish more widely. Future monitoring activities in these areas could be provided by expert's surveillance programs but directing the activities of citizen scientists toward these areas may be more effective and quicker. Promoting actively the collaborations between expert and citizens scientist can lead to the development and implementation of more effective monitoring programs for invasive species at a national scale.

## CONFLICT OF INTEREST

None declared.

## AUTHOR CONTRIBUTIONS

E.R.D., D.A.F., and B.D.C. conceived the idea. E.R.D. collected the data and led the analysis in collaboration with F.S., C.M. and D.A.F. Expert advice was provided by G.M., B.D.C. and T.C. E.R.D. drafted the manuscript in consultation with all authors.

## Data Availability

A digital version of the final ensemble model is provided on Figshare Digitial Repository (Roy‐Dufresne, Lurgi, et al., 2019; Roy‐Dufresne, Saltré, et al., 2019).
