# Supplementary material for: Modeling the distribution of a wide‐ranging invasive species using the sampling efforts of expert and citizen scientists
Source: Ecol Evol. 2019 Sep 19;9(19):11053–63. doi: 10.1002/ece3.5609 (PMC6802020; doi:10.1002/ece3.5609)
Supplement: Supplementary file 1 [file ECE3-9-11053-s001.docx]

**SUPPLEMENTARY MATERIALS FOR**

**Modelling the distribution of a wide-ranging invasive species using the sampling efforts of expert and citizen scientists.**

Emilie Roy-Dufresne^1*^, Frédérik Saltré^1,2^, Brian D. Cooke^3^, Camille Mellin^1,4^, Greg Mutze^5^, Tarnya Cox^6^, Damien A. Fordham^1^

^1^ The Environment Institute and School of Biological Sciences, University of Adelaide, Adelaide, SA, Australia

^2^ College of Science and Engineering, Flinders University, Adelaide, SA, Australia

^3^ Institute for Applied Ecology, University of Canberra, Canberra, ACT, Australia

^4^ Australian Institute of Marine Science, Townsville, QLD, Australia

^5^ Biosecurity SA, Department of Primary Industries and Regions South Australia, Adelaide, SA, Australia

^6^ Vertebrate Pest Research Unit, NSW Department of Primary Industries, Orange, NSW, Australia

*** CORRESPONDING AUTHOR:** Emilie Roy-Dufresne / roydufresne.emilie@gmail.com

**SUPPLEMENTARY MATERIAL S1**:
**Covariates selection and transformation**

We identified in Table S1 (from literature review and expert survey) 15 variables (i.e., 7 climate and 8 environmental variables) relevant to characterise the distribution of the rabbits across Australia.

**Table S1**: Summary of the initial list of variables analysed in the study.

| Category | Covariates | Description | References for justification and processing tasks |
| --- | --- | --- | --- |
| Climate | TMin^1^ | Annual 1976-2005 mean minimum temperature (°C) | Rabbits die if exposed to temperatures < -12°C (Myers et al., 1975) |
|  | TMax^1^ | Annual 1976-2005 mean maximum temperature (°C) | Rabbits die if exposed to temperature > 42°C (Kasa & Thwaites, 1990; Cooke et al., 2018) |
|  | TAvg^1^ | Annual 1976-2005 mean temperature (°C) | Relevant to vegetation growth. |
|  | TSea^1^ | Annual 1976-2005 mean of annual temperature seasonality (i.e. standard deviation*100) | Relevant to vegetation growth. |
|  | TWetQuarter^1^ | Annual 1976-2005 mean temperature of wettest quarter (mm) | Relevant to vegetation growth and warrens structure (Myers & Poole, 1963; Parer et al., 1987; Seltmann et al., 2009) |
|  | TWarmestMonth^1^ | Annual 1976-2005 mean of mean temperature of the warmest month (°C) | Rabbits start painting at 27°C and female rabbits stop lactating at 30°C (Gonzalez et al., 1971; Kluger, 2012) |
|  | AvgMonthTMinOver25^1^ | Annual 1976-2005 mean of number of months for which the monthly minimum temperature is greater than 25 °C | Rabbits start painting at 27°C and female rabbits stop lactating at 30°C (Gonzalez et al., 1971; Kluger, 2012). TMin was selected to see which regions, under the assumption that rabbits rest in warrens during the day, the rabbit will still undergo heat stress at night.  Information extracted from the monthly 1976-2005 minimum temperature. Using a R script we obtained the number of months for each year for which the minimum temperature was greater than 25°C, and averaged the results over the period of 1976-2005. |
|  | PWetQuarter^1^ | Annual 1976-2005 mean precipitation of wettest quarter (mm) | Relevant to vegetation growth and the percentage of water content in the pasture (Cooke, 1982; Cooke, Brennan, and Elsworth, 2018) |
|  | Prec^1^ | Annual 1976-2005 mean of annual precipitation (mm) | Relevant to vegetation growth, the percentage of water content in the pasture, and the risk for inundations (Myers & Poole, 1963; Cooke, 1982; Parer et al., 1987; Seltmann et al., 2009; Cooke, Brennan, and Elsworth, 2018) |
| Environment | VegeType^2^ | Major vegetation types for the Australian land (13 Classes) | The rabbits tend to avoid dense forest (Mallet and Cooke, 1986).  We reclassified the Major Vegetation Groups of the NVIS (Version 4.1) into 13 categories. The re-classification table in provided below. |
|  | DistPermWater^3,4^ | Euclidean weighted distance to permanent water features | Permanent water can be a good proxy source to find perennial vegetation and vegetation with greater percentage of water content (Myers & Poole, 1963; Cooke, 1982)  We calculated the Euclidean distance in km using ArcGIS 10.3.1 to any permanent water features as provided by the CSIRO and surface hydrology points (i.e. farm dam water, native well, water hole, and water tank) provided by Geoscience Australia. |
|  | DistAgriLand^5^ | Euclidean distance to agricultural land margins | Pasture is an important source of food for the rabbits (Cooke, 1982; Mallet and Cooke, 1986)  The data was calculated by sub-sampling the crop types (i.e. cropping, grazing irrigated modified pastures, irrigated cropping, perennial and seasonal horticulture, and irrigated perennial and seasonal horticulture, intensive horticulture, and intensive animal husbandry) within the land use dataset and by obtaining the Euclidean distance in km to them using ArcGIS 10.3.1. |
|  | PercSoilClay^6^ | Percentage of clay estimated at the A horizon | Can explain the distribution of the warrens in Australia and is a proxy for perennial vegetation (Myers & Parker, 1965; Hall & Myers, 1978) |
| Geography | MinDayLenght | Minimum day length across Australia | Daylength is a factor limiting the male rabbits’ reproduction period (Cooke, 1977; Boyd, 1985; Boyd, 1986).  Data obtained by calculating the day length across Australia using the geosphere package in R and by extracting the lowest value over a year for every site location. |
|  | VarDayLength | Variance in day lengths across Australia | Daylength is a factor limiting the male rabbits’ reproduction period (Cooke, 1977; Boyd, 1985; Boyd, 1986).  Data obtained by calculating the day length across Australia using the geosphere package in R and by calculating the variance over a year for every site location. |

^1^ Calculated or obtained from the eMAST database (<http://www.emast.org.au/>)

^2^ Data obtained from the Australian Major and Sub-Vegetation Groups dataset from the Environment Department of the Australian Government

^3^ Data obtained from CSIRO from the Atlas of Living Australia (<http://www.ala.org.au/>)

^4^ Data obtained from Geoscience Australia (http://www.ga.gov.au/)

^5^ Data from the Department of Agriculture of the Australian Government

^6^ Data obtained from the Australian Soil Resource Information System hosted by CSIRO

We reclassified the VegeType variable into 13 categories to reduce the number of factors included in the analysis (Fig. S1). (*i*) The first category is defined by the rainforest and vine thicket as it was in the initial variable. (*ii*) The second category now includes all eucalyptus forest (i.e. open forests, low open forests, open woodlands, and tropical woodlands/grasslands). (*iii*) The third category includes the eucalyptus woodland such as it was defined in the initial variable, and also mallee woodlands, mallee open woodlands, and sparse mallee shrublands. (*iv*) The fourth category is given the title ‘woodlands’ and includes all acacia forest, open acacia woodlands, callitris forests, casuarina forests, other forests and woodlands, and other open woodlands). (*v*) The fifth category englobe the seasonal inundated swamps, salt marshes and mangroves, which includes the initial mangroves, melaleuca forests, other grasslands, herblands, sedgelands, rushlands, and estuaries. (*vi*) The sixth category includes all low-closed forests and tall closed shrublands (including Acacia, Melaleuca and Banksia). (*vii*) The seventh category includes all shrublands, which are acacia, heathlands, and other shrublands. (*viii*) The eight category only includes the tussock grasslands as initial represented in the original variable. (*ix*) The neigh category is also similar to the original variable and only includes hummock grasslands. (*x*) The tenth category includes all chenopod shrublands, samphire shrublands, and forblands under the saltbushes category. (*xi*) The eleventh category encompasses all water features including inland aquatic, fresh water, salt lakes, estuaries, and lagoons. (*xii*) The category number twelve is defined by cleared vegetation, non-native vegetation, buildings, rocks, claypan, mudflat, and naturally bare areas. (*xiii*) The last category is defined by unclassified and unknown features.


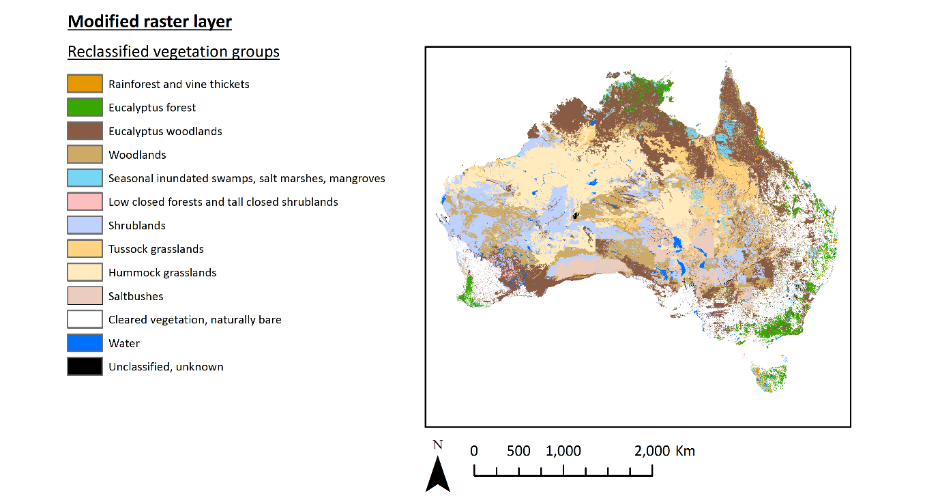


**Figure S1**: Raster categories included in the original ‘Vegetype’ raster layers and in the reclassified version.

We then tested for normality in the data and subsequently transformed some of the variables to reduce any potential nonlinearity of their response and improve the model performance (Austin 2002). Based on the results obtained, we made the following transformation on the variables: (*i*) square root for AvgMonthTMinOver25, DistAgriLand, DistPermWater, and VarDayLength, (*ii*) log for PercSoilClay, Prec, and PWetQuarter, and (*iii*) square for TAvg, TMax, and TWet. The other variables not previously listed were not transformed.

**References:**

Boyd, I. L. (1985) Effect of photoperiod and melatonin on testis development and regression in wild European rabbits (Oryctolagus cuniculus). Biology of reproduction, 33, 21-29.

Boyd, I. L. (1986) Photoperiod regulation of seasonal testicular regression in the wild European rabbit (Oryctolagus cuniculus). Journal of Reproduction and Fertility, 77, 463-470.

Cooke, B. D. (1977). Factors limiting the distribution of the wild rabbit in Australia. Proceedings of the Ecological Society of Australia - Volume10. Pp 113-120.

Cooke, B. D. (1982). A shortage of water in natural pastures as a factor limiting a population of rabbits, *Oryctolagus cuniculus* (L.), in Arid, North-Eastern South Australia. Australian Wildlife Research, 9, 465-476.

Cooke, B. D., Brennan, M., & Elsworth, P. (2018) Ability of wild rabbit, *Oryctolagus cuniculus*, to lactate successfully in hot environments explains continued spread in Australia’s monsoonal north. Wildlife Research, 45, 267-273.

Gonzalez, R. R., Kluger, M. J., & Hardy, J. D. (1971) Partitional calorimetry of the New Zealand white rabbit at temperatures 5°C-25°C. Journal of Applied Physiology, 31, 728-734.

Hall, L. S., & Myers, K. (1978). Variations in the microclimate in rabbit warrens in semi-arid New South Wales. Australian Journal of Ecology, 3(2), 187–194.

Kasa, W., & Thwaites, C. J. (1990). The effects of elevated temperature and humidity on rectal temperature and respiration rate in the New Zealand white rabbit. International Journal of Biometeorology, 34, 157-160.

Kluger, M. J. (2012). Energy balance in the resting and exercising rabbit. In Perspective in Biophysical Ecology (eds. D. M. Gates, & R. B. Schmerl.), pp. 496-507. Springer, Berlin.

Mallet, K. J., & Cooke, B. D. (1986). *The Ecology of the Common Wombat in South Australia*. Adelaide: Nature Conservation Society of South Australia.

Myers, K., & Parker, B. S. (1965). A study of the biology of the wild rabbit in climatically different regions in eastern Australia. CSIRO Wildlife Research, 10, 1-32.

Myers, K., Parker, B. S., & Dunsmore, J. D. (1975) Changes in numbers of rabbits and their burrows in a subalpine environment in South-estern New South Wales. Australia Wildlife Research, 2, 121-133.

Myers, K., & Poole, W. E. (1963). A study of the biology of the wild rabbit, *Oryctolagus cuniculus* (L.) in confined populations. IV The effets of rabbit grazing on sown pastures. Australian Journal of Ecology, 51, 431-451.

Parer, I., Fullagar, P. J., & Malafant, K. W. (1987). The history and structure of a large warren of the rabbit, *Oryctolagus cuniculus*, at Canberra, A.C.T. Australian Wildlife Research, 14, 505-513.

Seltmann, M. W., Ruf, T., & Rodel, H. G. (2009). Effects of body mass and huddling on resting metabolic rates of post-weaned European rabbits under different simulated weather conditions. Functional Ecology, 23, 1070-1080.

**SUPPLEMENTARY MATERIAL S2**:
**Sampling effort density grid and Ripley’s analysis.**


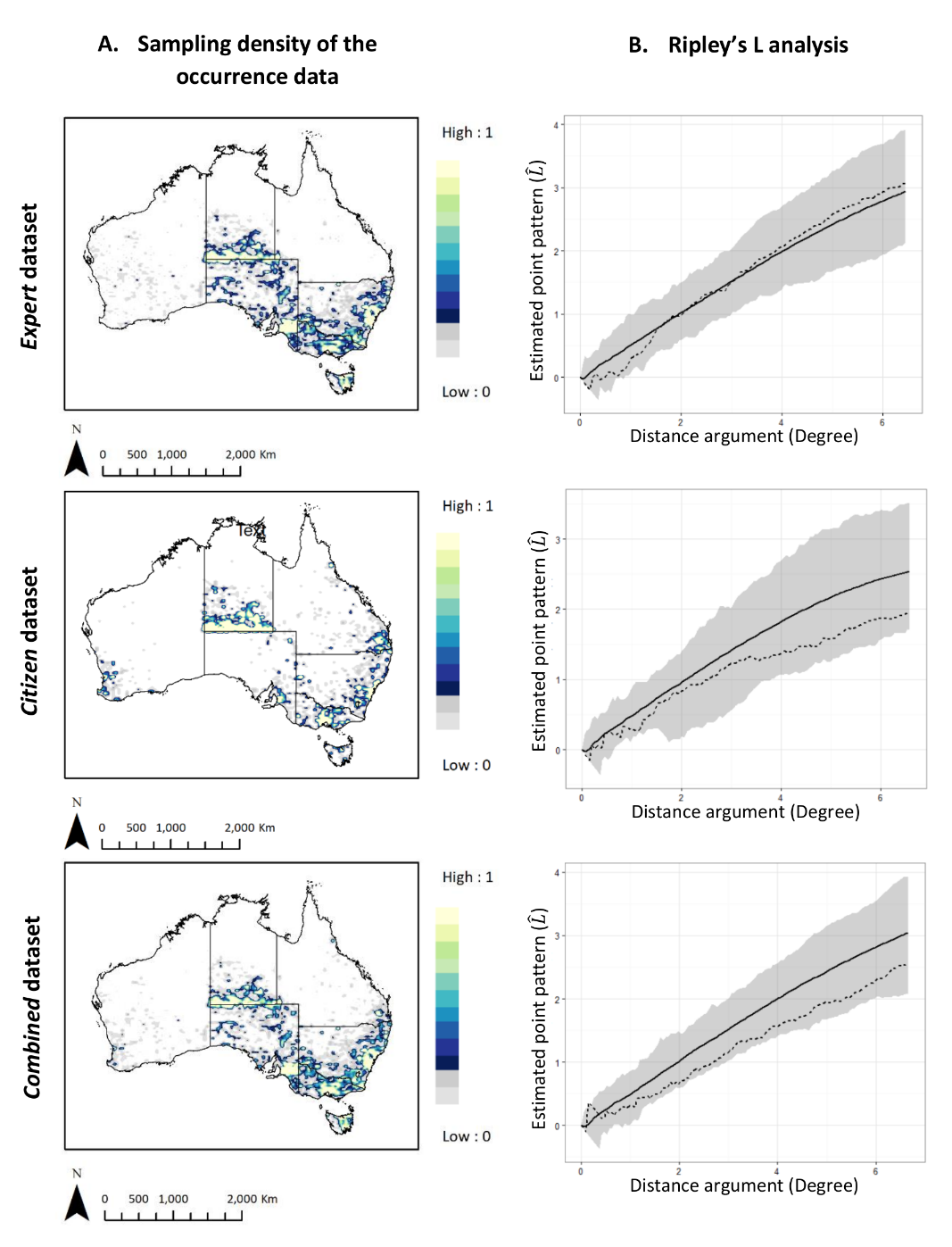


***Combined* dataset**

***Citizen* dataset**

***Expert* dataset**

**Figure S2**: Maps of the first column indicate the density of the occurrence data obtained by using the density function. The second column represents the results from the Ripley’s analysis so that the dotted line the calculated $\hat{L}$ value from the dataset, the solid line indicates the sample mean $\hat{L}$ from 10000 Monte-Carlo simulations assuming CSR (i.e., Complete Spatial Randomness) along with its 95% confidence interval (grey envelope). When the Ripley’s curves fall within the confidence level envelope, it indicates similar level of spatial density between the data used to generate the sampling grids and the pseudo-absences data. If the observed regression line goes above or below the 95% confidence envelope of the expected distribution, the observed data would described to be, respectively, more clustered or dispersed.

**SUPPLEMENTARY MATERIAL S3:**
**Testing for the degree of spatial auto-correlation in the occurrence data.**


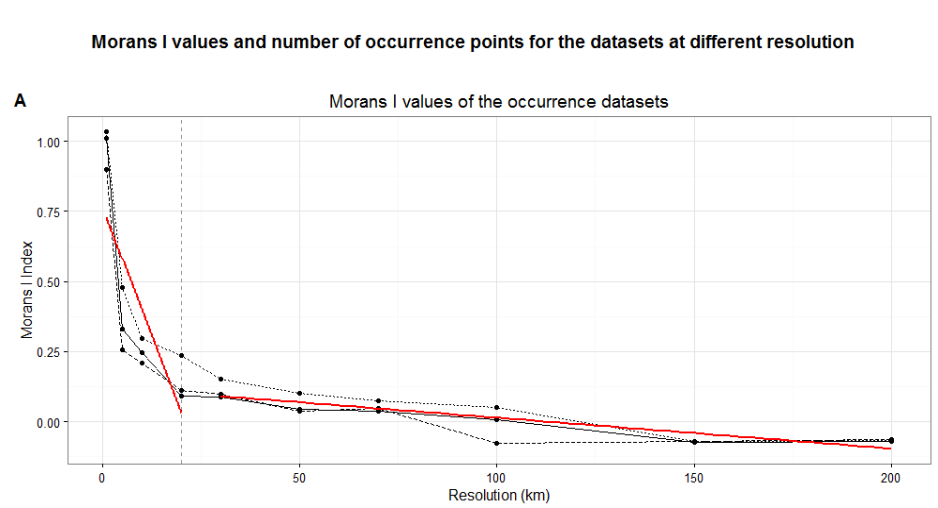


**Figure S3**: Moran’s I value for all datasets at different spatial resolution. The light grey dashed line highlights the value of the Moran’s I index at the selected 20km^2^ threshold and the red lines illustrate the difference in the slope of a regression equation above and below this threshold. The solid line represents the values for the *Expert* dataset, the small dashed line for the *Citizen* dataset, and the large dashed line for the *Combined* dataset.

**SUPPLEMENTARY MATERIAL S4**:
**Candidate models used in the analysis.**

**Table S4.1**: List of candidate models and their corresponding ecological hypothesis.

| Category of variables | Candidate models | Ecological hypothesis |
| --- | --- | --- |
| Climatic | ~ TMin | Rabbits avoid regions where the minimum temperature remains high at night. |
|  | ~ TWarmestMonth | Rabbits avoid regions where the monthly average maximum temperature remains higher than their biophysical tolerance. |
|  | ~ PWetQuarter | Rabbits avoid regions where the amount of precipitations is high which could flood their warrens. |
|  | ~ PWetQuarter + TWarmestMonth | Rabbits avoid humid regions which prevent them to dissipate heat stress. |
|  | ~ TMin + TWarmestMonth | Rabbits avoid really hot regions where temperature remains high at night and day for a month long. |
|  | ~ TMin + PWetQuarter | Rabbits avoid really hot regions where temperature remains high at night and where the amount of precipitations is important. |
|  | ~ TMin + TWarmestMonth + PWetQuarter | Rabbits avoid really hot regions where temperature remains high at night and day for a month long and where the amount of precipitations is important. |
| Environmental | ~ DistAgriLand | Rabbits remain close to agricultural land which provides green resources for food and water. |
|  | ~ DistPermWater | Rabbits remain close to permanent water resources to drink during heat waves. |
|  | ~ PercSoilClay | Rabbits select their habitat based on the underground substrate. |
|  | ~ VegeType | Rabbits live close to specific vegetation types which are proxy of good climate, food resources, and soil structure. |
|  | ~ DistAgriLand + VegeType | Rabbit’s distribution is mainly defined by access to green resources (i.e. for food and water). |
|  | ~ DistAgriLand + DistPermWater | Rabbit’s distribution is mainly defined by access to human related permanent water resources during hot seasons. |
|  | ~ VegeType + DistPermWater | Rabbit’s distribution is mainly defined by access to natural related permanent water resources during hot seasons. |
|  | ~ DistAgriLand + DistPermWater + PercSoilClay | Rabbit’s distribution is mainly defined by access to human related permanent water resources during hot seasons and good soil structure to construct a warren and hide during hot days. |
|  | ~ VegeType + DistPermWater + PercSoilClay | Rabbit’s distribution is mainly defined by access to natural related permanent water resources during hot seasons and good soil structure to construct a warren and hide during hot days. |
|  | ~ DistAgriLand + PercSoilClay | Rabbit’s distribution is mainly defined by interactions with human activities for food and water and good soil structure to construct a warren and hide during hot days. |
|  | ~ VegeType + PercSoilClay | Rabbit’s distribution is mainly defined by the surrounding vegetation type and good soil structure to construct a warren and hide during hot days. |
|  | ~ DistAgriLand + VegeType + PercSoilClay | Rabbit’s distribution is defined by all green resources for food and water and good soil structure to construct a warren and hide during hot days. |
|  | ~ DistAgriLand + VegeType + DistPermWater + PercSoilClay | Rabbit’s distribution is defined by all green resources for food and water, permanent water resources to drink when green resources are dry, and good soil structure to construct a warren and hide during hot days. |

**Table S4.2:** Table of the models results after testing all candidate models by only climatic or environmental variables, and the combination of both. The models were ranked using the Akaike’s information criterion corrected for small sample size (AICc), and each model probability was assessed relatively to the entire set of models using the AICc weights (wAICc) and the mean explained deviance.

|  | QuadGLM | | | | BRT | | | | MaxEnt | | | | | |
| --- | --- | --- | --- | --- | --- | --- | --- | --- | --- | --- | --- | --- | --- | --- |
| Pseudo-absences | *Random Pts* | | *Weighted Pts* | | *Random Pts* | | *Weighted Pts* | | *Random Pts* | | | *Weighted Pts* | | |
| Models | Mean wAIC_c_ | Mean explained deviance | Mean wAIC_c_ | Mean explained deviance | Mean wAIC_c_ | Mean explained deviance | Mean wAIC_c_ | Mean explained deviance | Mean wAIC_c_ | | Mean explained deviance | Mean wAIC_c_ | Mean explained deviance | |
| Climatic variables | | | | | | | | | | | | | |  |
| ~ TMin | 0.00 | 22.66 | 0.00 | 21.86 | 0.00 | 5.82 | 0.00 | 5.51 | - | - | | - | - |  |
| ~ TWarmestMonth | 0.00 | 12.68 | 0.00 | 12.23 | 0.00 | 21.91 | 0.00 | 21.11 | - | - | | - | - |  |
| ~ PWetQuarter | 0.00 | 5.14 | 0.00 | 4.83 | 0.00 | 22.91 | 0.00 | 22.09 | - | - | | - | - |  |
| ~ PWetQuarter + TWarmestMonth | 0.00 | 18.70 | 0.00 | 17.98 | 0.00 | 23.63 | 0.00 | 22.75 | 0.00 | 21.53 | | 0.00 | 20.79 |  |
| ~ TMin + TWarmestMonth | 0.00 | 23.53 | 0.00 | 22.67 | 0.00 | 25.28 | 0.00 | 24.45 | 0.00 | 26.72 | | 0.00 | 25.90 |  |
| ~ TMin + PWetQuarter | 0.00 | 22.77 | 0.00 | 21.94 | 1.00 | 27.29 | 1.00 | 26.30 | 1.00 | 24.63 | | 1.00 | 23.75 |  |
| ~ TMin + TWarmestMonth + PWetQuarter | 1.00 | 24.22 | 1.00 | 23.32 | 0.00 | 13.98 | 0.00 | 13.52 | 0.00 | 29.05 | | 0.00 | 28.08 |  |
| Other environmental variables | | | | | | | | | | | | | |  |
| ~ DistAgriLand | 0.00 | 4.37 | 0.00 | 4.27 | 0.00 | 5.54 | 0.00 | 5.20 | - | - | | - | - |  |
| ~ DistPermWater | 0.00 | 2.70 | 0.00 | 2.69 | 0.00 | 9.95 | 0.00 | 9.66 | - | - | | - | - |  |
| ~ PercSoilClay | 0.00 | 1.33 | 0.00 | 1.29 | 0.00 | 10.49 | 0.00 | 10.20 | - | - | | - | - |  |
| ~ VegeType | 0.00 | 0.19 | 0.00 | 0.17 | 0.00 | 3.43 | 0.00 | 3.33 | - | - | | - | - |  |
| ~ DistAgriLand + VegeType | 0.00 | 4.37 | 0.00 | 4.27 | 0.00 | 11.07 | 0.00 | 10.79 | 0.00 | 4.51 | | 0.00 | 4.39 |  |
| ~ DistAgriLand + DistPermWater | 0.00 | 5.70 | 0.00 | 5.60 | 0.00 | 5.66 | 0.00 | 4.32 | 0.00 | 6.44 | | 0.00 | 6.28 |  |
| ~ VegeType + DistPermWater | 0.00 | 2.92 | 0.00 | 2.88 | 0.00 | 10.92 | 0.00 | 10.43 | 0.00 | 5.37 | | 0.00 | 5.29 |  |
| ~ DistAgriLand + DistPermWater + PercSoilClay | 0.64 | 6.49 | 0.68 | 6.38 | 0.00 | 4.74 | 0.00 | 5.53 | 0.00 | 14.47 | | 0.00 | 14.10 |  |
| ~ VegeType + DistPermWater + PercSoilClay | 0.00 | 3.96 | 0.00 | 3.90 | 1.00 | 11.47 | 1.00 | 11.01 | 0.00 | 13.58 | | 0.00 | 13.24 |  |
| ~ DistAgriLand + PercSoilClay | 0.00 | 5.32 | 0.00 | 5.20 | 0.00 | 4.23 | 0.00 | 4.15 | 0.00 | 12.59 | | 0.00 | 12.24 |  |
| ~ VegeType + PercSoilClay | 0.00 | 1.63 | 0.00 | 1.57 | 0.00 | 10.54 | 0.00 | 10.04 | 1.00 | 11.87 | | 1.00 | 11.56 |  |
| ~ DistAgriLand + VegeType + PercSoilClay | 0.00 | 5.32 | 0.00 | 5.20 | 0.00 | 2.15 | 0.00 | 1.71 | 0.00 | 13.65 | | 0.00 | 13.28 |  |
| ~ DistAgriLand + VegeType + DistPermWater + PercSoilClay | 0.36 | 6.50 | 0.32 | 6.38 | 0.00 | 11.22 | 0.00 | 10.76 | 0.00 | 15.42 | | 0.00 | 15.03 |  |
| All Variables | | | | | | | | | | | | | |  |
| ~ All variables except PWetQuarter | 0.00 | 26.39 | 0.00 | 25.50 | 0.00 | 26.92 | 0.00 | 26.03 | 0.00 | 31.26 | | 0.00 | 30.36 |  |
| ~ All variables except PWetQuarter, DistAgriLand, and DistPermWater | 0.00 | 23.77 | 0.00 | 22.91 | 0.00 | 26.83 | 0.00 | 25.93 | 0.00 | 29.26 | | 0.00 | 28.40 |  |
| ~ All variables except PWetQuarter and VegeType | 0.00 | 26.38 | 0.00 | 25.49 | 0.20 | 26.90 | 0.00 | 26.03 | 0.00 | 31.03 | | 0.00 | 30.13 |  |
| ~ All variables except DistAgriLand and DistPermWater | 0.00 | 24.46 | 0.00 | 23.56 | 0.01 | 28.24 | 0.00 | 27.25 | 0.00 | 32.32 | | 0.00 | 31.31 |  |
| ~ All variables except Tmin | 0.00 | 24.36 | 0.00 | 23.52 | 0.00 | 24.77 | 0.00 | 23.89 | 0.00 | 30.91 | | 0.00 | 29.96 |  |
| ~ All variables except TMin, DistAgriLand, and DistPermwater | 0.00 | 18.89 | 0.00 | 18.17 | 0.00 | 23.96 | 0.00 | 23.10 | 0.00 | 27.55 | | 0.00 | 26.72 |  |
| ~ All variables except TMin and VegeType | 0.00 | 24.13 | 0.00 | 23.28 | 0.00 | 24.68 | 0.00 | 23.84 | 0.00 | 30.34 | | 0.00 | 29.42 |  |
| ~ All variables except TWarmestMonth, DistAgriLand, and DistPermWater | 0.00 | 23.12 | 0.00 | 22.29 | 0.00 | 24.94 | 0.00 | 24.01 | 0.00 | 29.00 | | 0.00 | 28.06 |  |
| ~ All variables except TWarmestMonth | 0.00 | 26.85 | 0.00 | 25.91 | 0.00 | 25.19 | 0.00 | 24.26 | 0.00 | 31.34 | | 0.00 | 30.35 |  |
| ~ All variables except TWarmestMonth and VegeType | 0.00 | 26.72 | 0.00 | 25.77 | 0.00 | 25.16 | 0.00 | 24.25 | 0.00 | 30.83 | | 0.00 | 29.83 |  |
| ~ All variables except VegeType | 0.00 | 28.02 | 0.00 | 27.04 | 0.20 | 28.28 | 0.66 | 27.31 | 0.00 | 33.65 | | 0.00 | 32.61 |  |
| ~ All variables | 0.99 | 28.13 | 0.99 | 27.16 | 0.79 | 28.30 | 0.33 | 27.30 | 1.00 | 34.00 | | 1.00 | 32.93 |  |

**SUPPLEMENTARY MATERIAL S5**:
**Models response curves**

**Table S5.1**: Theoretical model response curves according to the rabbit’s ecology.

| Variables name | Figures | Ecological explanation |
| --- | --- | --- |
| TMin | 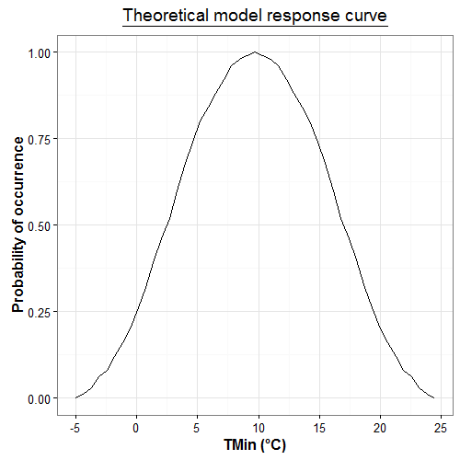 | When the temperature minimum is below the freezing threshold, the frost can harm or kill plants and damages crops. Such events prevent the rabbits to have access to food resources, and therefore limit their occurrence in regions where frost events are common (Myers and Parker 1965, Dunsmore 1974).  As the ambient temperature raises during the day, rabbits spend most of the day underneath the ground in their warrens where the ambient temperature is cooler (Hall and Myers 1978), or rest in the shade created by bushes (Wood, personal communication). Shortly before sunset, when the ground surface temperature becomes cooler, the rabbits emerge from the burrows to feed. However, in regions where the temperature at night remains greater than 25°C, female lactating rabbits remains under a constant stress factor. According to Cooke (unpublished), Parigi-Bini *et al.* (1992), and Tablado *et al.* (2008), there is little effective breeding by the rabbits when the ambient temperature exceed 25°C. |
| TWarmestMonth | 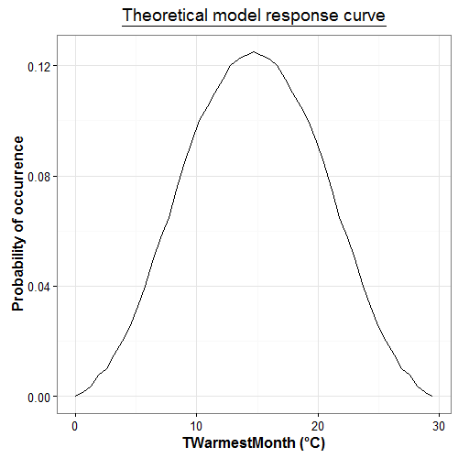 | Rabbits are able to dissipate heat in hot conditions by altering the blood flow in their ear (Kluger 2012) and by increasing their respiration rates. They however start to stress when the ambient temperature exceeds 27°C in dry atmosphere and face lethal body temperature when it rise towards 42°C (Cooke, unpublished). |
| PWetQuarter | 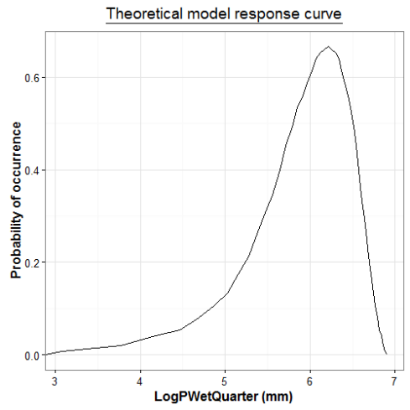 | An increase in rain induces the growth of green vegetation on the Australian land, which is often followed by an increase of the rabbit’s population (Myers and Parker 1975, Wood 1980, Cooke 1983, Wheeler and King 1985, Wells *et al.* 2006). According to some technical reports, the rabbits can breed and survive during the hot summer months in addition to winter months as long as green vegetation is present (Rabbit Biology and Control In Arid and Semi-Arid Lands).  An excessive amount of rain can however lead to mortality due to the flooding of the warrens (Parker 1977). In addition, Kasa and Thwaites (1990) showed that in tropical regions, higher level of humidity reduces the capacity of the rabbits to dissipate heat through evaporation, increasing the stress related to over-heating. |
| VegeType | 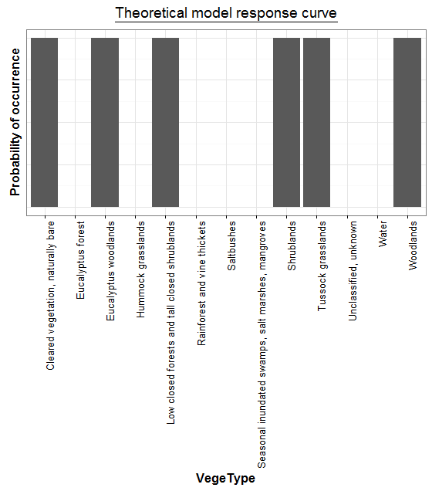 | The type of vegetation observed across Australia influences the distribution of the rabbits on the basis that vegetation provides the food and water resources essential for the rabbits to survive (Myers and Parker 1975). Martin *et al.* (2007) described the diet of the rabbits as being quite flexible along the seasons, but that in all circumstances, rabbits preferred to consume plant species with high water content (>54%) to help to maintain their intake of water. In winter, the species relies greatly on pasture species. As the summer die-off these plants resources, the rabbits then shift to seeds, succulent and drought-resistant perennial vegetation (Cooke 1982, Martin *et al.* 2007). Regions where the water content in the vegetation is low or not accessible to rabbits are therefore often free of rabbit populations, such as reported for the spinifex regions in the Northern Territory (Rabbit range reduction NT final, 1998) and forested areas (Dellafore *et al.* 2008). |
| DistPermWater | 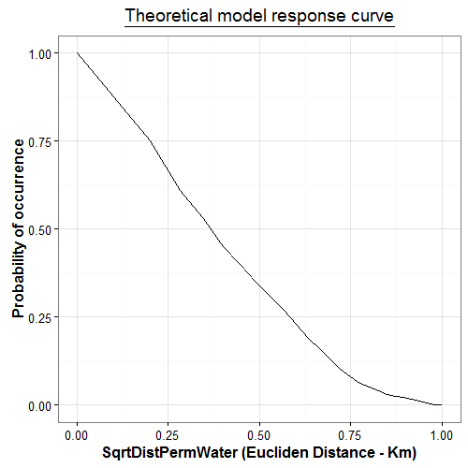 | Rabbits are known to primarily rely on the water content of the plants they consume to maintain their water balance. During drought period however, shortage of water in the vegetation forces the rabbits to seek for other options. During this critical period, rabbits can climb into trees to obtain succulent leaves and twigs, or are observed to drink at permanent water springs and water troughs (Cooke 1982). According to Cooke (1982) and Berman *et al.* (2006, 2011), rabbits that are provided access to water during droughts maintain their weight and have a greater survival probability than those which are not given this access. |
| DistAgriLand | 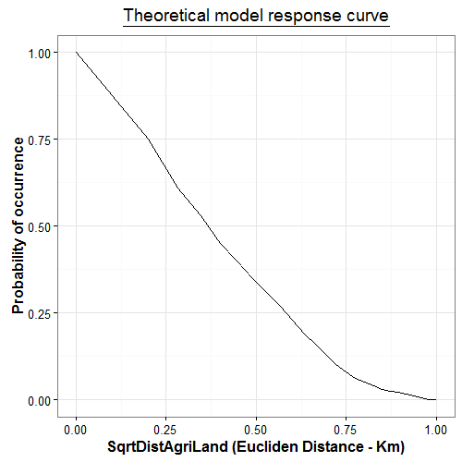 | Pasture crops are a mix of annual and perennial grasses and are rich in proteins and water content. They provides essential food resources to the rabbits and insure the survival of its populations. The new growth of pasture crops after the dry season is also commonly reported to induce the reproduction process in the rabbit populations (Myers and Parker 1965, Hughes and Rowley 1966). On the other hand, results from a simulation model showed that a decrease of 15% of the pasture growth reduce the population size by 0-50% (Richards 1979, Scanlan *et al.* 2006). |
| PercSoilClay | 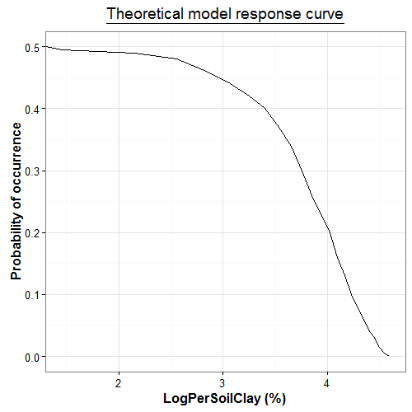 | The occurrence and survival probability of the species differ between the land systems in Australia, especially in arid areas. According to Myers and Parker (1975), the distribution of the rabbit’s warrens is in close relationship to the drainage patterns of the soil. In regions with a lower level of clay in the soil, rabbits populations rapidly decrease in number after a drought, whereas in regions with higher clay content (e.g. close to large swamps), rabbits populations were observed to survive due to the availability of water and the repeated germinations of perennials plants (Myers and Parker 1975). If the level of clay is too high, however, the ability of the rabbits to dig their warrens in the soil decreases and the solidity of the structure of those warrens, under important rain, decreases (Parer and Parker 1986, Berman *et al.* 2011).  In addition, the percentage of clay in the soil also influences the microclimate of the warrens that is the temperature and humidity within the burrows (Hall and Myers 1978). Such characteristics affect the rabbit’s body temperature regulation and water conservation, which are critical aspects that allow the rabbits to survive in extreme hot conditions (Hayward 1961). Greater level of clay content in the soil enables the temperature and humidity within the burrow to remain steady while both measures respectively increases and decreases rapidly at the ground surface level (Hall and Myers 1978). Rabbits can reduce their water requirement by up to 40% by breathing the cool humid air within the burrow (Hayward 1961). |

**:**

**Table S5.2**: Evaluation criteria for the models’ response curves.

| Grade | Corresponding criteria |
| --- | --- |
| 0 | The response curve of the model does not correspond at all to what expected from the biology of the species. |
| 1 | The general shape of the response curve follows what is expected according to the biology of the species. |
| 2 | The general shape of the response curve follows what is expected according to the biology of the species. In addition, the height of the curve illustrates well the expected influence of the variable on the probability to observe the species. |
| 3 | The general shape of the response curve follows what is expected according to the biology of the species. In addition, the height of the curve illustrates well the expected influence of the variable on the probability to observe the species. Finally, the starting and ending values associated with the different fluctuations observed in the curve correspond to known biophysical limitation threshold of the species. |

**SUPPLEMENTARY MATERIAL S6**:
**Results from the predictive accuracy analysis at continental and regional scale.**

**Table S6.1**: Summary of the mean values and standard deviation values (from the replicates) for the predictive accuracy measures, AUC and Kappa, and the resulting weighted scores from the response curves analysis for models calibrated with *Random Pts* pseudo-absences.

| Datasets | Pseudo-absence strategy | Model algorithms | | AUC | | KAPPA | | Response curves weighted scores |
| --- | --- | --- | --- | --- | --- | --- | --- | --- |
|  |  |  |  | Mean | SD | Mean | SD |  |
| *Expert* | *Random Pts* | | BRT | 0.892 | 0.004 | 0.512 | 0.009 | 40.34 |
|  |  | | MaxEnt | 0.869 | 0.001 | 0.456 | 0.004 | 31.01 |
|  |  | | Quadratic GLM | 0.849 | 0.001 | 0.437 | 0.003 | 36.64 |
|  | *Weighted Pts* | | BRT | 0.887 | 0.001 | 0.500 | 0.003 | 40.27 |
|  |  | | MaxEnt | 0.864 | 0.001 | 0.445 | 0.004 | 29.6 |
|  |  | | Quadratic GLM | 0.844 | 0.001 | 0.431 | 0.003 | 34.82 |
| *Citizen* | *Random Pts* | | BRT | 0.888 | 0.002 | 0.494 | 0.006 | 25.3 |
|  |  | | MaxEnt | 0.870 | 0.003 | 0.465 | 0.007 | 39.72 |
|  |  | | Quadratic GLM | 0.829 | 0.002 | 0.453 | 0.006 | 21.08 |
|  | *Weighted Pts* | | BRT | 0.888 | 0.002 | 0.488 | 0.007 | 25.22 |
|  |  | | MaxEnt | 0.869 | 0.002 | 0.464 | 0.007 | 36.53 |
|  |  | | Quadratic GLM | 0.829 | 0.002 | 0.423 | 0.006 | 22.36 |
| *Combined* | *Random Pts* | | BRT | 0.886 | 0.001 | 0.540 | 0.004 | 39.37 |
|  |  | | MaxEnt | 0.880 | 0.002 | 0.525 | 0.004 | 28.97 |
|  |  | | Quadratic GLM | 0.845 | 0.001 | 0.465 | 0.003 | 38.42 |
|  | *Weighted Pts* | | BRT | 0.880 | 0.001 | 0.524 | 0.004 | 36.75 |
|  |  | | MaxEnt | 0.858 | 0.001 | 0.480 | 0.003 | 30.79 |
|  |  | | Quadratic GLM | 0.839 | 0.001 | 0.455 | 0.003 | 37.81 |

**Table S6.2**: Summary of the Kappa mean values by regions, the range of values for the overall regions, and the standard deviations among the scores obtained for all regions for models calibrated with *Random Pts* pseudo-absences.

| Dataset | Pseudo-absences strategy | Models algorithms | Regional mean value | | | | | | | | | | | | | | Min value | Max value | SD |
| --- | --- | --- | --- | --- | --- | --- | --- | --- | --- | --- | --- | --- | --- | --- | --- | --- | --- | --- | --- |
|  |  |  | #3 | #5 | #7 | #8 | #9 | #12 | #13 | #16 | #17 | #18 | #19 | #20 | #21 | #23 |  |  |  |
| *Expert* | *Random Pts* | BRT | 0.26 | 0.26 | 0 | 0.41 | 0 | 0.17 | 0.25 | 0.06 | 0 | 0.4 | 0.07 | 0 | 0.25 | 0 | 0 | 0.41 | 0.15 |
|  |  | MaxEnt | 0.04 | 0.03 | 0 | 0.33 | 0 | 0.24 | 0.21 | 0.05 | 0 | 0.38 | 0.08 | 0 | 0.21 | 0 | 0 | 0.38 | 0.13 |
|  |  | Quadratic GLM | 0.1 | 0.02 | 0 | 0.34 | 0 | 0.15 | 0.21 | 0 | 0 | 0.33 | 0 | 0 | 0.24 | 0 | 0 | 0.34 | 0.13 |
|  | *Weighted Pts* | BRT | 0.13 | 0.16 | 0 | 0.37 | 0 | 0.19 | 0.2 | 0.06 | 0.01 | 0.34 | 0.07 | 0 | 0.15 | 0.02 | 0 | 0.37 | 0.12 |
|  |  | MaxEnt | 0.04 | 0.03 | 0 | 0.32 | 0 | 0.24 | 0.21 | 0.04 | 0 | 0.37 | 0.07 | 0 | 0.17 | 0.01 | 0 | 0.37 | 0.12 |
|  |  | Quadratic GLM | 0.06 | 0.01 | 0 | 0.27 | 0 | 0.16 | 0.11 | 0 | 0.01 | 0.35 | 0 | 0 | 0.18 | 0 | 0 | 0.35 | 0.11 |
| *Citizen* | *Random Pts* | BRT | 0.13 | 0.14 | 0.11 | 0.12 | 0 | 0.15 | 0.2 | 0.53 | 0.02 | 0.26 | 0.07 | 0.39 | 0.3 | 0.11 | 0 | 0.53 | 0.14 |
|  |  | MaxEnt | 0.1 | 0.1 | 0.12 | 0.15 | 0.02 | 0.18 | 0.22 | 0.54 | 0.06 | 0.13 | 0.05 | 0.39 | 0.31 | 0.17 | 0.02 | 0.54 | 0.14 |
|  |  | Quadratic GLM | 0.08 | 0.12 | 0.13 | 0.14 | 0 | 0.12 | 0.21 | 0.38 | 0.01 | 0.11 | 0.02 | 0.38 | 0.32 | 0.11 | 0 | 0.38 | 0.12 |
|  | *Weighted Pts* | BRT | 0.13 | 0.14 | 0.11 | 0.12 | 0 | 0.15 | 0.2 | 0.52 | 0.02 | 0.25 | 0.07 | 0.39 | 0.29 | 0.11 | 0 | 0.52 | 0.14 |
|  |  | MaxEnt | 0.11 | 0.11 | 0.12 | 0.15 | 0.02 | 0.21 | 0.22 | 0.53 | 0.06 | 0.14 | 0.05 | 0.39 | 0.31 | 0.16 | 0.02 | 0.53 | 0.14 |
|  |  | Quadratic GLM | 0.08 | 0.07 | 0.12 | 0.14 | 0 | 0.1 | 0.19 | 0.5 | 0.05 | 0.16 | 0.02 | 0.37 | 0.32 | 0.13 | 0 | 0.5 | 0.14 |
| *Combined* | *Random Pts* | BRT | 0.09 | 0.12 | 0.11 | 0.26 | 0 | 0.19 | 0.12 | 0.24 | 0.03 | 0.32 | 0.09 | 0.04 | 0.12 | 0.02 | 0 | 0.32 | 0.09 |
|  |  | MaxEnt | 0.03 | 0.01 | 0.07 | 0.28 | 0 | 0.21 | 0.2 | 0.25 | 0 | 0.34 | 0.11 | 0.1 | 0.13 | 0.01 | 0 | 0.34 | 0.11 |
|  |  | Quadratic GLM | 0.06 | 0.02 | 0.08 | 0.22 | 0 | 0.09 | 0.17 | 0.16 | 0.01 | 0.35 | 0 | 0.08 | 0.18 | 0 | 0 | 0.35 | 0.1 |
|  | *Weighted Pts* | BRT | 0.08 | 0.12 | 0.1 | 0.25 | 0 | 0.18 | 0.11 | 0.22 | 0.02 | 0.31 | 0.1 | 0.03 | 0.11 | 0.01 | 0 | 0.31 | 0.09 |
|  |  | MaxEnt | 0.03 | 0.01 | 0.06 | 0.27 | 0 | 0.21 | 0.18 | 0.24 | 0 | 0.34 | 0.12 | 0.07 | 0.1 | 0.02 | 0 | 0.34 | 0.11 |
|  |  | Quadratic GLM | 0.03 | 0 | 0.05 | 0.17 | 0 | 0.12 | 0.05 | 0.17 | 0.01 | 0.35 | 0.01 | 0.04 | 0.1 | 0 | 0 | 0.35 | 0.09 |

**Table S6.3**: Summary of the AUC mean values by regions, the range of values for the overall regions, and the standard deviations among the scores obtained for all regions for models calibrated with *Random Pts* pseudo-absences.

| Dataset | Pseudo-absences strategy | Models algorithms | Regional mean value | | | | | | | | | | | | | | Min value | Max value | SD |
| --- | --- | --- | --- | --- | --- | --- | --- | --- | --- | --- | --- | --- | --- | --- | --- | --- | --- | --- | --- |
|  |  |  | #3 | #5 | #7 | #8 | #9 | #12 | #13 | #16 | #17 | #18 | #19 | #20 | #21 | #23 |  |  |  |
| *Expert* | *Random Pts* | BRT | 0.71 | 0.72 | 0.39 | 0.81 | 0.5 | 0.69 | 0.67 | 0.59 | 0.78 | 0.73 | 0.67 | 0.46 | 0.67 | 0.52 | 0.39 | 0.81 | 0.12 |
|  |  | MaxEnt | 0.65 | 0.8 | 0.38 | 0.77 | 0.46 | 0.73 | 0.65 | 0.56 | 0.77 | 0.73 | 0.66 | 0.43 | 0.65 | 0.53 | 0.38 | 0.80 | 0.13 |
|  |  | Quadratic GLM | 0.69 | 0.64 | 0.39 | 0.72 | 0.45 | 0.63 | 0.64 | 0.57 | 0.76 | 0.71 | 0.53 | 0.45 | 0.63 | 0.44 | 0.39 | 0.76 | 0.11 |
|  | *Weighted Pts* | BRT | 0.7 | 0.72 | 0.39 | 0.8 | 0.51 | 0.68 | 0.67 | 0.58 | 0.77 | 0.73 | 0.66 | 0.46 | 0.66 | 0.52 | 0.39 | 0.80 | 0.12 |
|  |  | MaxEnt | 0.65 | 0.8 | 0.38 | 0.76 | 0.46 | 0.73 | 0.65 | 0.56 | 0.76 | 0.72 | 0.66 | 0.43 | 0.64 | 0.54 | 0.38 | 0.80 | 0.13 |
|  |  | Quadratic GLM | 0.69 | 0.64 | 0.39 | 0.71 | 0.45 | 0.63 | 0.64 | 0.57 | 0.75 | 0.71 | 0.53 | 0.45 | 0.63 | 0.45 | 0.39 | 0.75 | 0.11 |
| *Citizen* | *Random Pts* | BRT | 0.65 | 0.68 | 0.68 | 0.71 | 0.67 | 0.55 | 0.68 | 0.9 | 0.78 | 0.68 | 0.84 | 0.86 | 0.7 | 0.69 | 0.55 | 0.90 | 0.09 |
|  |  | MaxEnt | 0.6 | 0.71 | 0.64 | 0.64 | 0.67 | 0.61 | 0.69 | 0.87 | 0.72 | 0.67 | 0.77 | 0.87 | 0.72 | 0.72 | 0.60 | 0.87 | 0.08 |
|  |  | Quadratic GLM | 0.56 | 0.7 | 0.64 | 0.59 | 0.57 | 0.52 | 0.66 | 0.89 | 0.74 | 0.64 | 0.66 | 0.84 | 0.71 | 0.57 | 0.52 | 0.89 | 0.10 |
|  | *Weighted Pts* | BRT | 0.66 | 0.69 | 0.68 | 0.71 | 0.68 | 0.55 | 0.68 | 0.9 | 0.78 | 0.67 | 0.84 | 0.86 | 0.7 | 0.68 | 0.55 | 0.90 | 0.09 |
|  |  | MaxEnt | 0.61 | 0.71 | 0.64 | 0.64 | 0.67 | 0.63 | 0.69 | 0.87 | 0.71 | 0.66 | 0.77 | 0.87 | 0.72 | 0.71 | 0.61 | 0.87 | 0.08 |
|  |  | Quadratic GLM | 0.56 | 0.69 | 0.64 | 0.6 | 0.58 | 0.52 | 0.66 | 0.88 | 0.74 | 0.64 | 0.65 | 0.84 | 0.72 | 0.56 | 0.52 | 0.88 | 0.10 |
| *Combined* | *Random Pts* | BRT | 0.72 | 0.8 | 0.64 | 0.78 | 0.53 | 0.66 | 0.67 | 0.7 | 0.76 | 0.73 | 0.67 | 0.59 | 0.64 | 0.51 | 0.51 | 0.80 | 0.08 |
|  |  | MaxEnt | 0.61 | 0.8 | 0.57 | 0.72 | 0.47 | 0.67 | 0.67 | 0.69 | 0.73 | 0.72 | 0.69 | 0.59 | 0.61 | 0.54 | 0.47 | 0.80 | 0.08 |
|  |  | Quadratic GLM | 0.67 | 0.67 | 0.58 | 0.68 | 0.47 | 0.58 | 0.67 | 0.69 | 0.72 | 0.71 | 0.53 | 0.59 | 0.64 | 0.46 | 0.46 | 0.72 | 0.08 |
|  | *Weighted Pts* | BRT | 0.71 | 0.78 | 0.63 | 0.77 | 0.52 | 0.66 | 0.66 | 0.69 | 0.75 | 0.72 | 0.68 | 0.58 | 0.64 | 0.51 | 0.51 | 0.78 | 0.08 |
|  |  | MaxEnt | 0.61 | 0.8 | 0.56 | 0.72 | 0.47 | 0.68 | 0.66 | 0.68 | 0.72 | 0.71 | 0.7 | 0.58 | 0.59 | 0.54 | 0.47 | 0.80 | 0.09 |
|  |  | Quadratic GLM | 0.67 | 0.67 | 0.57 | 0.67 | 0.46 | 0.59 | 0.66 | 0.68 | 0.72 | 0.71 | 0.54 | 0.58 | 0.63 | 0.46 | 0.46 | 0.72 | 0.08 |

**Table S6.4** Number of physiographic regions per models with *Random* *Pts* or *Weighted Pts* pseudo-absence with greater AUC and Kappa scores using when compared according to their occurrence datasets (i.e. *Expert*. *Citizen*. or *Combined*). The results were obtained from the out-of-region analyses.

| Models | Pseudo-absences strategy | Number of regions with greater AUC score | | |  | Number of regions with greater Kappa score | | | |
| --- | --- | --- | --- | --- | --- | --- | --- | --- | --- |
|  |  | *Expert* | *Citizen* | *Combined* |  | | *Expert* | *Citizen* | *Combined* |
| BRT | *Random Pts* | 4 | 7 | 0 |  | 3 | | 9 | 2 |
| MaxEnt |  | 3 | 13 | 1 |  | 5 | | 8 | 0 |
| Quadratic GLM |  | 2 | 12 | 0 |  | 4 | | 8 | 0 |
| BRT | *Weighted Pts* | 6 | 5 | 3 |  | 2 | | 8 | 2 |
| MaxEnt |  | 3 | 13 | 1 |  | 5 | | 8 | 0 |
| Quadratic GLM |  | 4 | 8 | 1 |  | 4 | | 8 | 1 |

No attribution was given when the region had the same highest score for two different datasets.


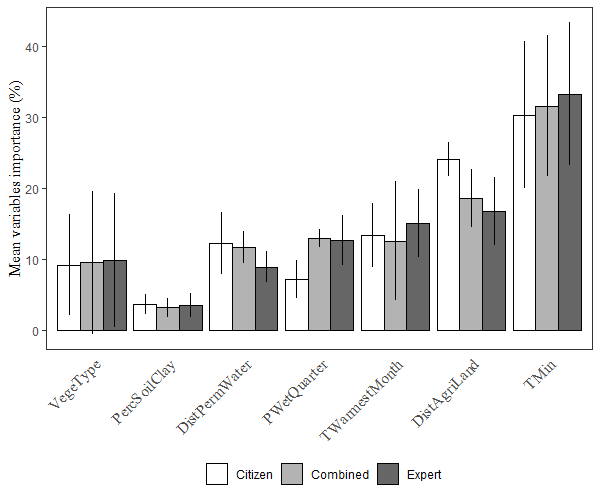


**Figure S6.1:** Mean of the variables importance (%) and their corresponding standard deviations (line range) for the *Weighted* *Pts* pseudo-absence strategy based on three different sources of dataset (i.e. *Expert*, *Citizen*, and *Combined*).

*
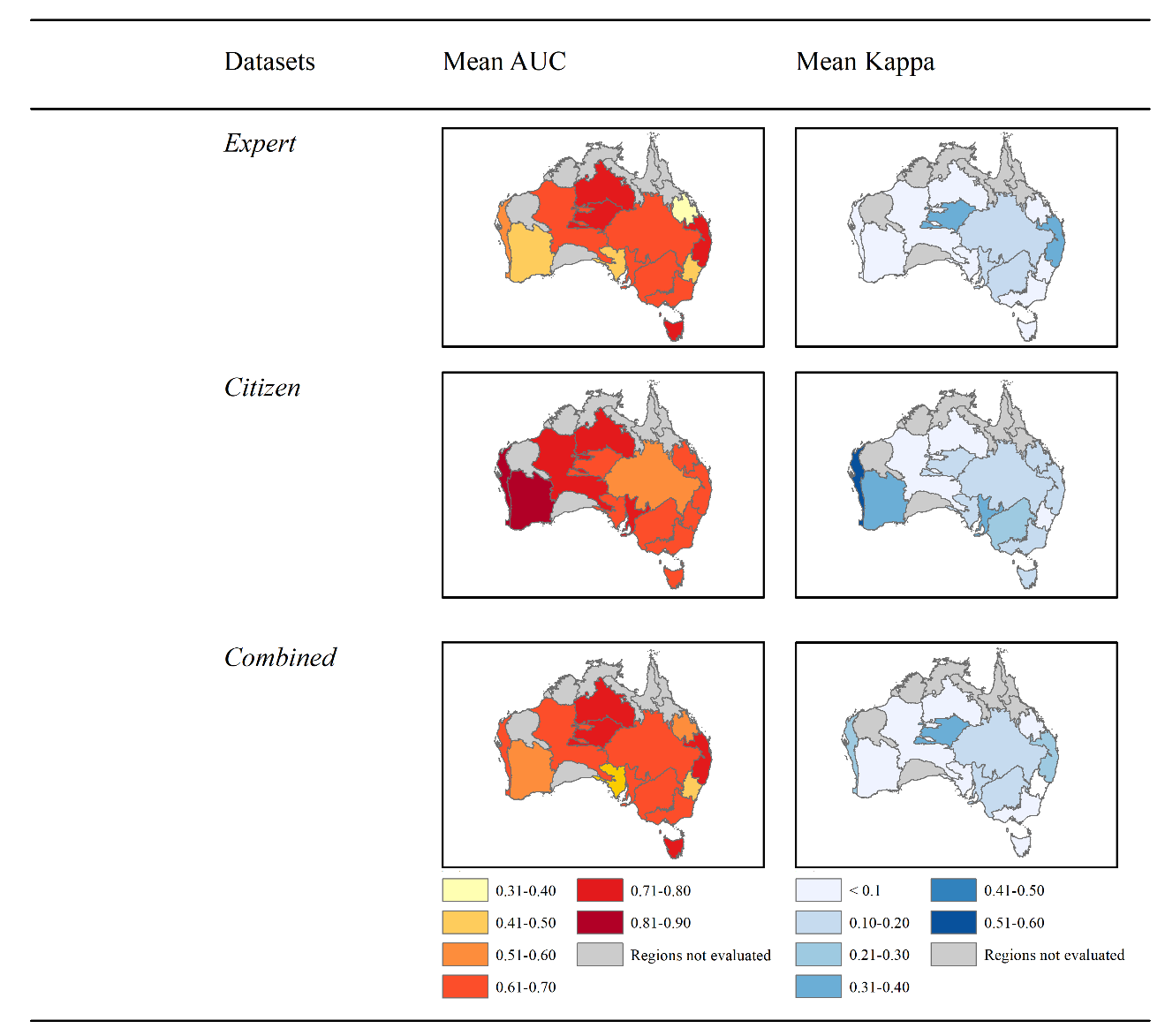
*

**Figure S6.2**: AUC and Kappa results from the out-of-regions analyses based on three different sources of dataset (i.e. *Expert,* *Citizen*, and *Combined*) for the *Weighted Pts* pseudo-absence strategy. The figures were obtained by taking the mean of the results across all algorithms. The land divisions represent the locations of the physiographic regions of Australia and the regions in grey were not evaluated due to low number of occurrence points (n<25).

**SUPPLEMENTARY MATERIAL S7**:
**Qualitative visual examination of the response curves from the Experts.**


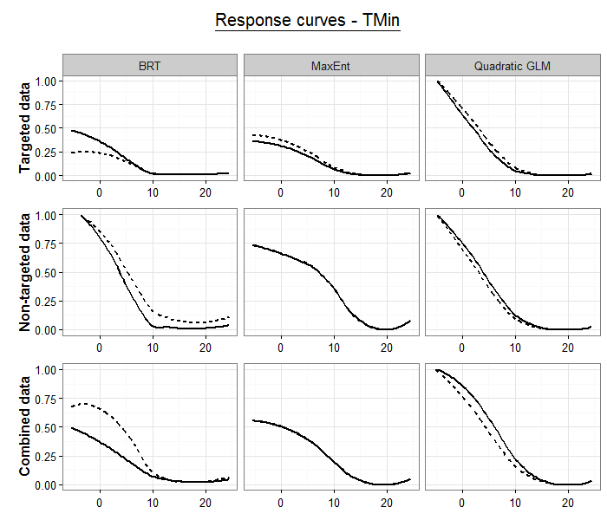


**TMin (°C)**

***Combined* data**

***Citizen* data**

***Expert* data**

**Figure S7.1**: Response curves obtain for the variable *TMin* for all models. The full lines represent the *Random* *Pts* pseudo-absences, and the dashed lines the *WeightedPts* pseudo-absences.


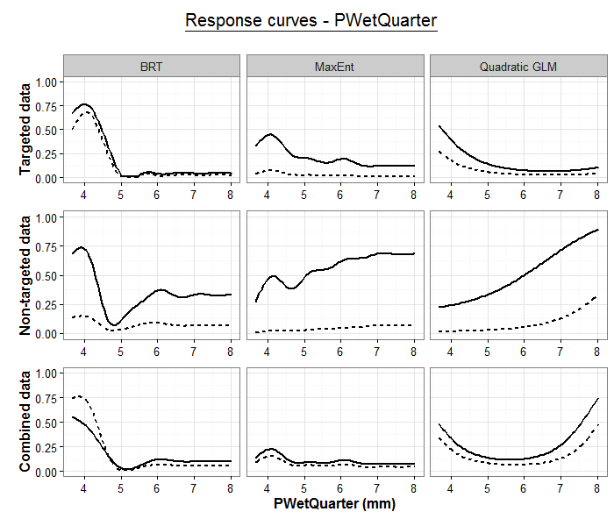


***Combined* data**

***Expert* data**

***Citizen* data**

**Figure S7.2**: Response curves obtain for the variable *PWetQuarter* for all models. The full lines represent the *Random* *Pts* pseudo-absences, and the dashed lines the *WeightedPts* pseudo-absences.


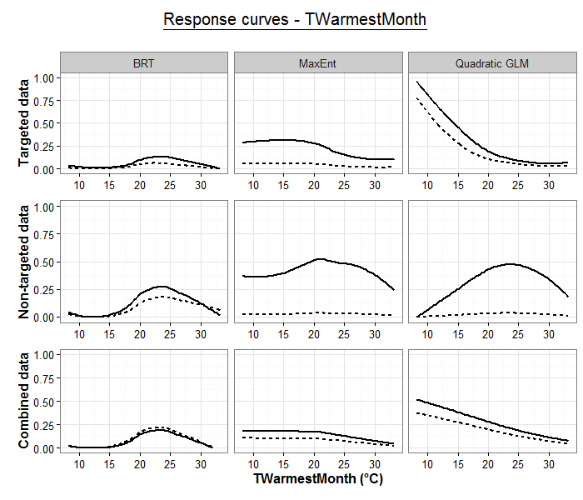


***Combined* data**

***Citizen* data**

***Expert* data**

**Figure S7.3**: Response curves obtain for the variable *TWarmestMonth* for all models. The full lines represent the *Random* *Pts* pseudo-absences, and the dashed lines the *WeightedPts* pseudo-absences.


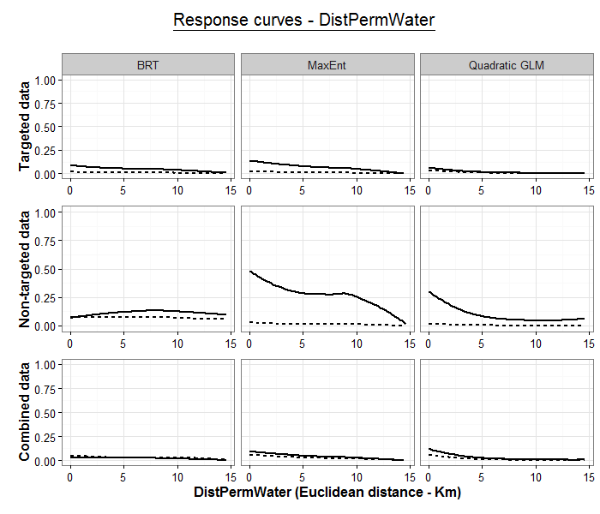


***Combined* data**

***Citizen* data**

***Expert* data**

**Figure S7.4**: Response curves obtain for the variable *DistPermWater* for all models. The full lines represent the *Random* *Pts* pseudo-absences, and the dashed lines the *WeightedPts* pseudo-absences.


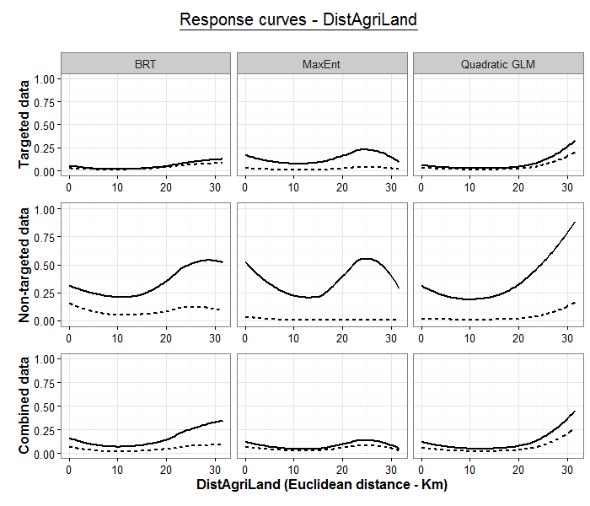


***Combined* data**

***Expert* data**

***Citizen* data**

**Figure S7.5**: Response curves obtain for the variable *DistAgriLand* for all models. The full lines represent the *Random* *Pts* pseudo-absences, and the dashed lines the *WeightedPts* pseudo-absences.


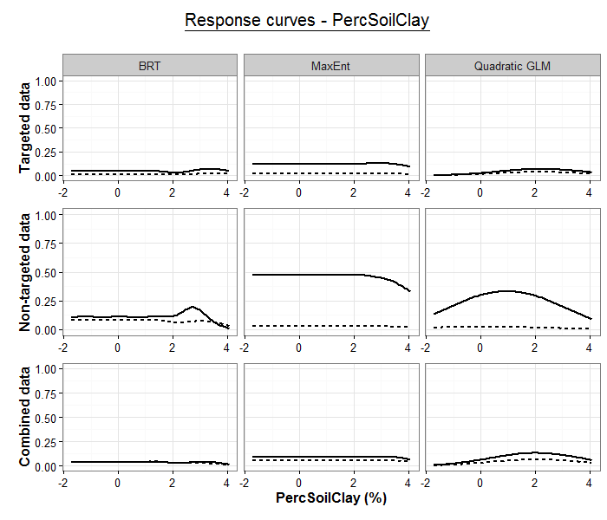


***Combined* data**

***Citizen* data**

***Expert* data**

**Figure S7.6**: Response curves obtain for the variable *PercSoilClay* for all models. The full lines represent the *Random* *Pts* pseudo-absences, and the dashed lines the *WeightedPts* pseudo-absences.


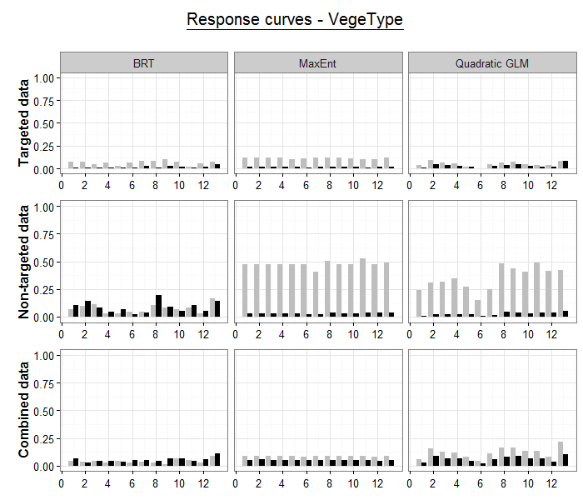


***Combined* data**

***Citizen* data**

***Expert* data**

**Figure S7.7**: Response curves obtain for the variable *VegeType* for all models. The grey bars represent the *Random* *Pts* pseudo-absences, and the black bars the *WeightedPts* pseudo-absences.

**SUPPLEMENTARY MATERIAL S8**:
**Results from individual models used to generate the ensemble model.**

**
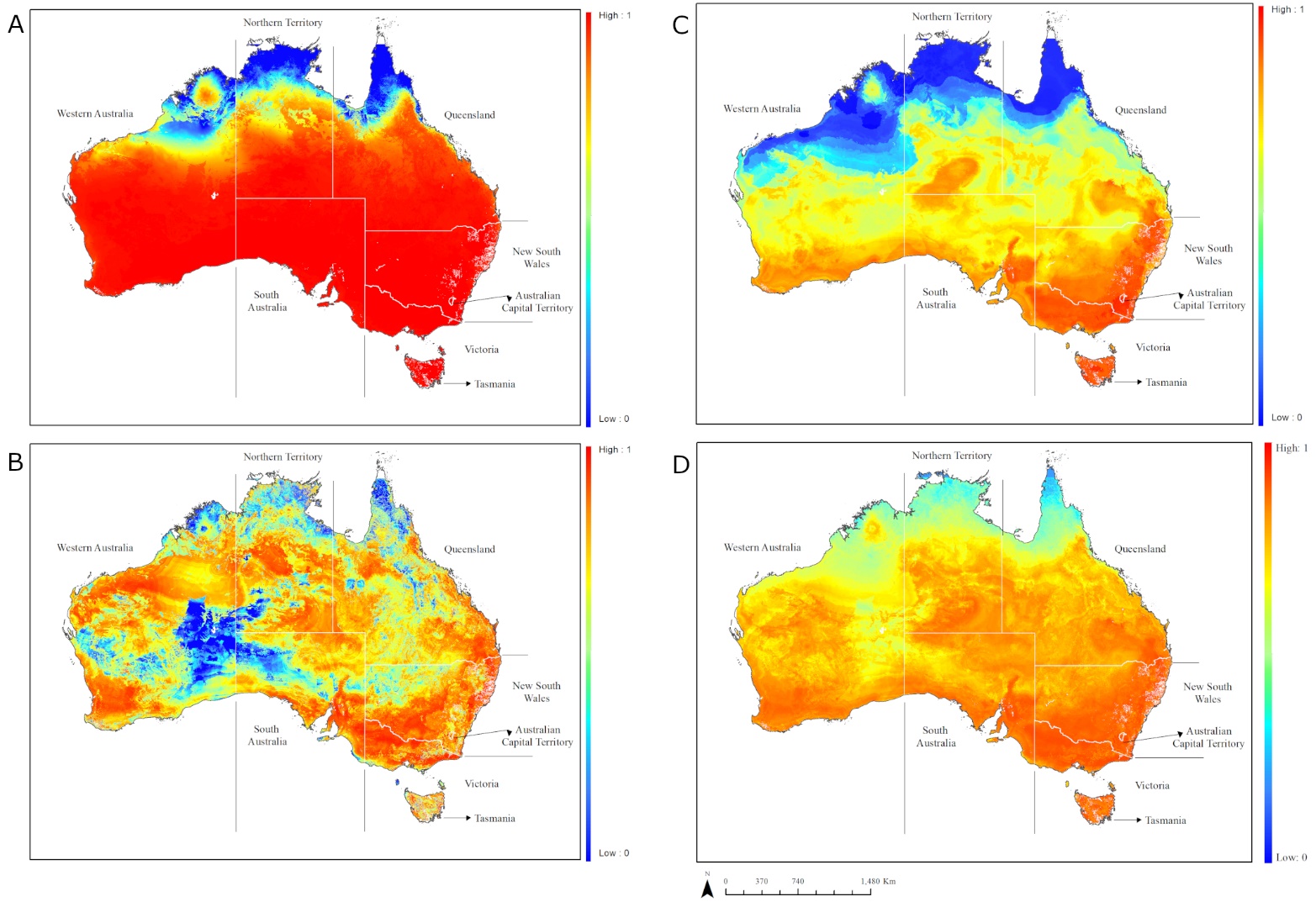
**

**Figure S8.1**: Probability of occurrence of rabbits across Australia according to the (A) GLM, (B) BRT, (C) MaxEnt model and (D) ensemble model. Gradient goes from dark blue (probability 0) to bright red (probability of 1). The white land divisions and the dotted lines represent the location of state boundaries in Australian.
